# Biologic TNF-α inhibitors reduce microgliosis, neuronal loss, and tau phosphorylation in a transgenic mouse model of tauopathy

**DOI:** 10.1186/s12974-021-02332-7

**Published:** 2021-12-31

**Authors:** Weijun Ou, Joshua Yang, Juste Simanauskaite, Matthew Choi, Demi M. Castellanos, Rudy Chang, Jiahong Sun, Nataraj Jagadeesan, Karen D. Parfitt, David H. Cribbs, Rachita K. Sumbria

**Affiliations:** 1 Department of Biomedical and Pharmaceutical Sciences, School of Pharmacy, Chapman University, Irvine, CA 92618 USA; 2 Henry E. Riggs School of Applied Life Sciences, Keck Graduate Institute, Claremont, CA 91711 USA; 3 Department of Neuroscience, Pomona College, Claremont, CA 91711 USA; 4 Keck Science Department, Claremont McKenna College, Claremont, CA 91711 USA; 5 MIND Institute, University of California, Irvine, CA 92697 USA; 6 Department of Neurology, University of California, Irvine, CA 92868 USA

**Keywords:** Biologic TNF-α inhibitor, Alzheimer’s disease, Tau, Microgliosis, Transferrin receptor, Molecular Trojan Horse, Blood–brain barrier

## Abstract

**Background:**

Tumor necrosis factor-α (TNF-α) plays a central role in Alzheimer’s disease (AD) pathology, making biologic TNF-α inhibitors (TNFIs), including etanercept, viable therapeutics for AD. The protective effects of biologic TNFIs on AD hallmark pathology (Aβ deposition and tau pathology) have been demonstrated. However, the effects of biologic TNFIs on Aβ-independent tau pathology have not been reported. Existing biologic TNFIs do not cross the blood–brain barrier (BBB), therefore we engineered a BBB-penetrating biologic TNFI by fusing the extracellular domain of the type-II human TNF-α receptor (TNFR) to a transferrin receptor antibody (TfRMAb) that ferries the TNFR into the brain via receptor-mediated transcytosis. The present study aimed to investigate the effects of TfRMAb-TNFR (BBB-penetrating TNFI) and etanercept (non-BBB-penetrating TNFI) in the PS19 transgenic mouse model of tauopathy.

**Methods:**

Six-month-old male and female PS19 mice were injected intraperitoneally with saline (*n* = 12), TfRMAb-TNFR (1.75 mg/kg, *n* = 10) or etanercept (0.875 mg/kg, equimolar dose of TNFR, *n* = 10) 3 days/week for 8 weeks. Age-matched littermate wild-type mice served as additional controls. Blood was collected at baseline and 8 weeks for a complete blood count. Locomotion hyperactivity was assessed by the open-field paradigm. Brains were examined for phosphorylated tau lesions (Ser202, Thr205), microgliosis, and neuronal health. The plasma pharmacokinetics were evaluated following a single intraperitoneal injection of 0.875 mg/kg etanercept or 1.75 mg/kg TfRMAb-TNFR or 1.75 mg/kg chronic TfRMAb-TNFR dosing for 4 weeks.

**Results:**

Etanercept significantly reduced phosphorylated tau and microgliosis in the PS19 mouse brains of both sexes, while TfRMAb-TNFR significantly reduced these parameters in the female PS19 mice. Both TfRMAb-TNFR and etanercept treatment improved neuronal health by significantly increasing PSD95 expression and attenuating hippocampal neuron loss in the PS19 mice. The locomotion hyperactivity in the male PS19 mice was suppressed by chronic etanercept treatment. Equimolar dosing resulted in eightfold lower plasma exposure of the TfRMAb-TNFR compared with etanercept. The hematological profiles remained largely stable following chronic biologic TNFI dosing except for a significant increase in platelets with etanercept.

**Conclusion:**

Both TfRMAb-TNFR (BBB-penetrating) and non-BBB-penetrating (etanercept) biologic TNFIs showed therapeutic effects in the PS19 mouse model of tauopathy.

**Supplementary Information:**

The online version contains supplementary material available at 10.1186/s12974-021-02332-7.

## Introduction

Alzheimer’s disease (AD) is a progressive neurodegenerative disease that was first discovered in 1906 by the German clinical psychiatrist and neuroanatomist, Aloïs Alzheimer [1]. The neuropathological alterations reported by Alzheimer in 1906 constitute the main pathological hallmarks of AD today: extracellular deposition of amyloid-beta (Aβ)-containing plaques and intracellular tau-containing neurofibrillary tangles in the brain [2]. While the accumulation of Aβ plaques in the brain is a characteristic of AD pathology, abnormal intracellular accumulation of tau lesions is observed in several neurodegenerative disorders, known as tauopathies [3].

Apart from the predominant role of Aβ and tau pathology in AD, neuroinflammation has emerged as a driver and an accelerator of AD pathology [4–6], and therefore provides a broad window of opportunity (from early to late-stage AD) for therapeutic intervention. Among the pro-inflammatory mediators, TNF-α is one of the main inflammatory cytokines involved in initiating and propagating an inflammatory response [7]. Both clinical and preclinical studies support the role of peripheral and central nervous system (CNS) TNF-α in AD, and elevated TNF-α levels were observed in the serum [8, 9] and postmortem brains of AD patients and AD mouse models [10, 11]. Furthermore, serum TNF-α levels correlated with disease progression in AD patients [9]. Global TNF-α receptor 1 (TNFR-1) knock-out prevented Aβ generation and learning deficits in the APP23 AD mice [12]. Direct application of TNF-α to cell culture preparations, or CNS administration of TNF-α, increased Aβ production, tau phosphorylation, and synaptic dysfunction [13–17], and accelerated disease progression and cognitive decline [18]. Similarly, peripheral TNF-α modulated AD pathology in mouse models [19, 20]. Adding to this, TNF-α polymorphisms linked with increased TNF-α production were found to be associated with late-onset AD [21].

Several studies report the effects of the existing FDA-approved biologic TNF-α inhibitors on AD pathology both in humans and preclinical models. For example, infliximab, a chimeric anti-TNF-α antibody, significantly reduced cognitive deficits in a female AD patient and significantly reduced Aβ- and tau-pathology and memory impairment in AD mouse models, following intrathecal and intracerebroventricular administration [22–24], respectively. Similarly, perispinal etanercept, a fusion protein of the extracellular domain of the type-II TNFR and human IgG1 Fc-domain, improved cognitive deficits in AD patients [25]. Accordingly, intrahippocampal injection of dominant-negative TNF-α inhibitors reduced Aβ pathology in the 3xTg mouse model [26]. Recently, intracerebroventricular injection of a nanobody directed against the TNFR-1 was protective in two mouse models of amyloidosis [27]. One commonality among the aforementioned studies is the invasive route of injection used to evaluate the CNS effects of these biologic TNF-α inhibitors due to their limited blood–brain barrier (BBB) penetration. For example, the brain uptake of etanercept was found to be equal to that of a human IgG control [28].

Tau protein belongs to the family of microtubule-associated proteins, has a high binding affinity for microtubules, and plays an important role in microtubule stabilization for optimal neuronal function [29]. Tauopathies, including AD, are characterized by the aggregation of hyperphosphorylated tau protein, which is strongly correlated with neuroinflammation [30, 31]. Recent studies have shown that microglial activation and release of pro-inflammatory cytokines exacerbate tau pathology, independent of Aβ pathology [31, 32], and that anti-inflammatory strategies can reduce tau pathology in mice [33, 34]. Despite a large body of literature looking at the effects of TNF-α inhibition on AD pathology, the effects of TNF-α inhibition on Aβ-independent tau pathology have not been reported.

To study the effects of direct CNS TNF-α blockage via a non-invasive injection route, we engineered a BBB-penetrating biologic TNF-α inhibitor by fusing the extracellular domain of the type-II TNFR (decoy TNFR) to a transferrin receptor antibody (TfRMAb) for BBB penetration [35, 36]. The TfRMAb-TNFR fusion protein binds to the BBB TfR and enters the brain via receptor-mediated transcytosis. The resulting brain concentrations of the TfRMAb-TNFR are > 40-fold higher than that of a non-TfR targeting IgG [36]. Our previous work showed that this BBB-penetrating TNF-α inhibitor reduces Aβ plaques, insoluble Aβ1-42, BBB-disruption, brain endothelial activation, and cognitive memory deficits in the APP/PS1 double transgenic AD mouse model of amyloidosis [37]. Overall, the BBB-penetrating TNF-α inhibitor had better therapeutic indices than etanercept, a non-BBB-penetrating TNF-α inhibitor [28] expected to modulate only peripheral but not CNS TNF-α, in the mouse model of amyloidosis [37]. However, the effects of these biologic TNF-α inhibitors on tau pathology are currently unknown.

Based on the above, the goal of the current investigation was to examine the effects of peripheral and CNS TNF-α inhibition on tau pathology using the P301S mutant tau transgenic mouse model of tauopathy (line PS19). For this, we first evaluated the effect of the biologic TNF-α inhibitors (etanercept: non-BBB-penetrating and TfRMAb-TNFR: BBB-penetrating) on tau phosphorylation, microgliosis, and neuronal health. We further assessed the effect of the biologic TNF-α inhibitors on the hyperactive phenotype of the PS19 mice using the open-field test. To gain insight into the differential therapeutic effects of the two biologic inhibitors, we examined the plasma pharmacokinetics of these biologics in mice. Finally, the overall hematologic safety of chronic biologic TNF-α inhibitor dosing was evaluated by performing a complete blood count in the PS19 mice.

## Methods

### Fusion protein

TfRMAb-TNFR was produced via transient expression in CHO-K1 cells, and cell supernatants were collected for protein A purification followed by size exclusion chromatography (WuXi Biologics, NJ, USA), as described previously [36]. The final protein purity was > 96%, and the target protein was verified by Western blot. Etanercept was obtained from International Laboratory USA (CA, USA). The affinity of the TfRMAb-TNFR fusion protein to the mouse TfR and human TNF-α was confirmed through ELISA (Additional file 1: Supplemental Methods, Additional file 2: Fig. S1A, B). TNF-α inhibitory effects were confirmed in vitro using induced pluripotent stem cell (iPSC)-derived human brain microvascular endothelial cells (ihBMECs; Additional file 1: Supplemental Methods, Additional file 2: Fig. S1C, D). Both the TfRMAb-TNFR fusion protein and etanercept were formulated in 100 mM glycine, 150 mM NaCl, 28 mM Tris, and 0.01% Polysorbate 80, pH = 6.42, sterile filtered, and stored at − 80 °C until use.

### Chronic dosing in a mouse model of tauopathy

**Fig. 1:**
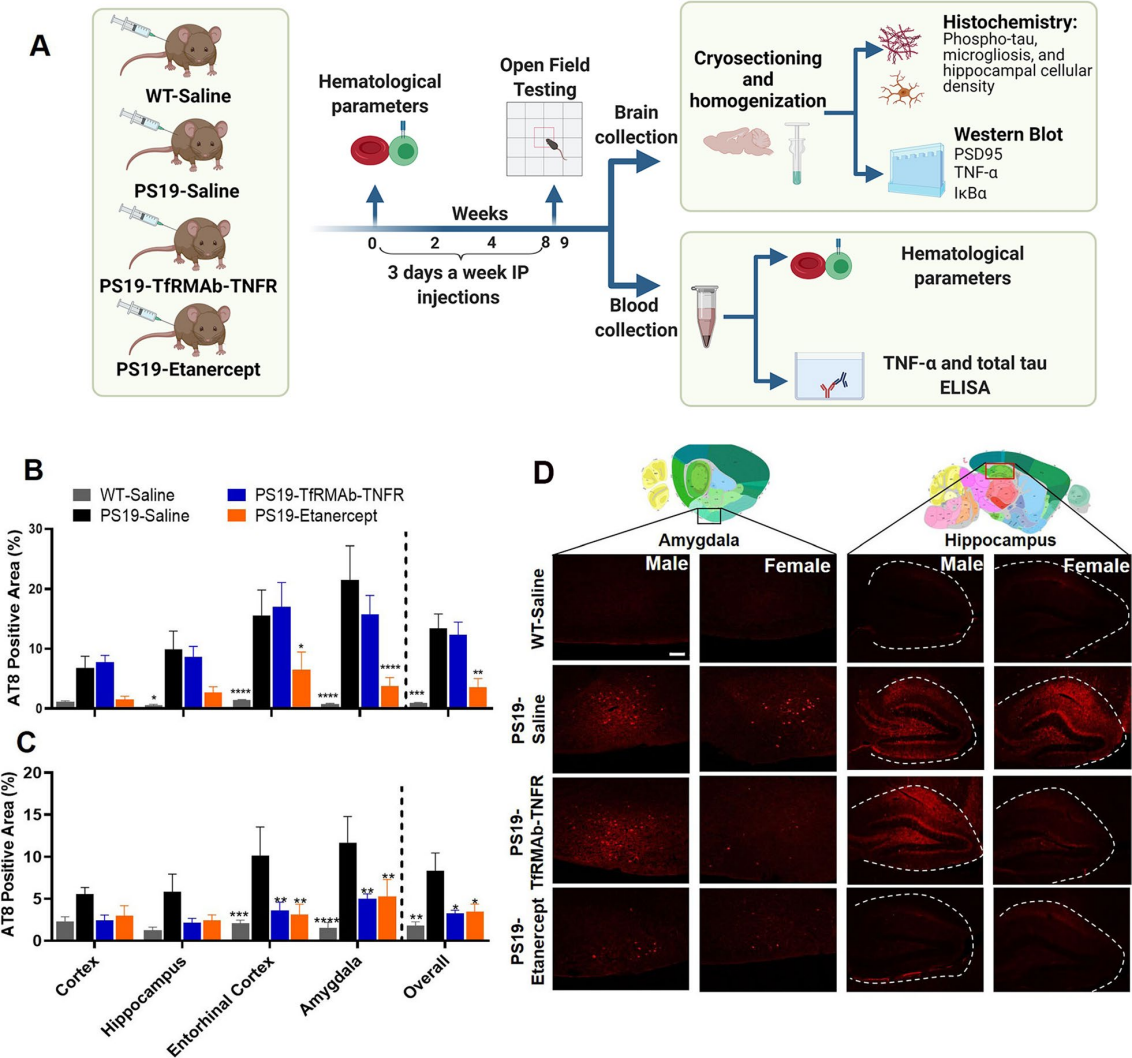
Effect of TNF-α inhibitors on pTau (Ser202, Thr205) in the PS19 mice. Schematic showing the experimental timeline for treatment and sample processing of the PS19 mice. Figure was prepared using BioRender.com (**A**). AT8-positive area in different brain regions in male (**B**) and female (**C**) mice. The overall AT8-positive area, which is an average of the regional AT8-positive area, was significantly reduced by etanercept in both sexes and by TfRMAb-TNFR in female mice (**B**, **C**). Representative images of AT8-positive pTau in PS19 mice (**D**). The thumbnail brain section images showing the hippocampus (red box) and amygdala (black box) in **D** are taken from Allen Institute. Scale bar = 150 μm. Data are presented as mean ± SEM of 10–12 mice per treatment group (5–7 per sex per group). Two-way ANOVA with repeated measures with Holm–Sidak’s post hoc test was used to compare to PS19-Saline controls. *p < 0.05, **p < 0.01, ***p < 0.001, ****p < 0.0001

Animal studies were performed on hemizygous Tg(Prnp-MAPT*P301S)PS19Vle (PS19) mice (6 months old at the start of the study, Jackson Laboratories, ME, USA) in compliance with University Laboratory Animal Resources under protocols approved by the University of California, Irvine, Institutional Animal Care and Use Committee. Mice were provided constant access to food and water and were maintained under a 12 h light/12 h dark cycle. Mice were injected intraperitoneally (IP) three days per week for 8 weeks with saline (PS19-Saline; *n* = 12, female = 7, male = 5), TfRMAb-TNFR (PS19-TfRMAb-TNFR; 1.75 mg/kg, *n* = 10, female = 5, male = 5), or etanercept (PS19-Etanercept; 0.875 mg/kg, *n* = 10, female = 5, male = 5). These are equimolar doses of TfRMAb-TNFR and etanercept since the TNFR domain comprises ~ 50% of etanercept and ~ 26% of the TfRMAb-TNFR fusion protein based on amino acid sequence [36, 38]. Age-matched noncarrier wild-type (WT) littermates (*n* = 12, female = 6, male = 6) received equivalent volume of saline via the IP route (Fig. 1A). All the mice were evaluated for signs of immune response (general appearance, spontaneous locomotion, and posture) after each injection, and body weights were checked weekly. Blood was collected at baseline and 8 weeks after treatment for a complete blood count (Molecular Diagnostic Services, Inc., CA, USA). At the end of 8 weeks, mice were subjected to the open-field paradigm that was performed over a week, anesthetized with a lethal dose of Euthasol (150 mg/kg, IP), and perfused with ice-cold phosphate buffer saline (PBS). Brains were harvested, and hemi-brains were fixed in 4% paraformaldehyde (PFA) for immunostaining or were frozen in dry ice for Western blotting (Fig. 1A).

### Open-field testing

Since the PS19 mice consistently demonstrate a hyperactivity phenotype compared to cognitive deficits [39–41], we used the open-field test to measure locomotion hyperactivity. The open-field test was performed after 8 weeks of treatment (during week 9) as described previously [42–44]. Briefly, mouse movements were recorded for 5 min after placing in a white open box (72 cm x 72 cm with 36 cm walls). Mean speed and total distance were measured for locomotion evaluation. A center square, measuring 36 cm × 36 cm was drawn in the open box to measure time in the center. Time in the center was used as a measure of anxiety in mice. All analysis was performed using the SMART 3.0 Video Tracking Software (Panlab, Harvard Apparatus, MA, USA).

### Cryosectioning

The right-cerebral hemi-brain of each mouse was immersion-fixed with 4% PFA in PBS for 24 h, followed by serial incubation in 10%, 20%, and 30% sucrose solution at 4 °C for 24 h each, which was followed by freezing. The frozen brain tissues were mounted in Tissue-Tek OCT compound (Fisher Scientific, MA, USA), and sliced into 20-μm-thick sagittal sections at − 25 °C using a cryostat (Micron Instruments, CA, USA). Five sections (600 µm apart) per mouse were used for immunostaining and histology as described below.

### Phospho-tau (pTau; Ser202, Thr205) and Iba-1 immunostaining

Free-floating sagittal brain sections were washed three times for 2 min in PBS and blocked with 0.5% bovine serum albumin (BSA) in PBS containing 0.3% Triton X-100 (TX100) for 60 min at room temperature. Adjacent tissue sections were incubated with either 0.2% biotin-conjugated pTau monoclonal antibody (AT8 antibody to detect tau phosphorylated at ser202, thr205; Thermo Fisher Scientific, MA, USA) or 0.5 µg/mL anti-Iba-1 rabbit antibody (to detect microgliosis) in PBS containing 0.3% TX100 and 0.5% BSA overnight at 4 °C. The tissue sections were then washed three times in PBS for 5 min and incubated in the dark with 0.5% AlexaFluor 594 conjugated streptavidin antibody (Biolegend; CA, USA) for pTau detection or with 0.1% AlexaFluor 488 donkey anti-rabbit IgG (Biolegend; CA, USA) in PBS containing 0.3% TX-100 and 0.5% BSA for 2 h at room temperature. The sections were then washed three times in PBS for 10 min and quickly dipped in distilled water to remove the salts. The sections were mounted onto glass slides, cover-slipped with Vectamount aqueous mounting media (Vector Laboratories, CA, USA), and sealed with nail polish. Slides were stored at 4 °C until imaging.

### pTau (Ser202, Thr205) and Iba-1 quantification

The fluorescent staining was analyzed using a Leica TCS SP5 Confocal Microscope (Leica, NJ, USA). For immunostaining, five distinct brain sections (600 µm apart) for each mouse were used for analysis. For each brain section, two regions in the cerebral cortex, one or two regions in the hippocampus, one region in the entorhinal cortex, and one region in the amygdala were examined and imaged at a 10 × magnification. For Iba-1 immunostaining, for each brain section, three regions in the cortex, two regions in the hippocampus, two regions in the entorhinal cortex, and one region in the amygdala was imaged at a 10 × magnification with 3 × digital zoom. The digitized images were analyzed using the NIH ImageJ (version 1.53e, MD, USA) using a threshold setting to calculate tissue area positive for the AT8 or Iba-1. For this, immunofluorescent images were converted to 16-bit and inverted to grayscale. Threshold values of images were manually calibrated to eliminate background noise. After adjusting the threshold, the “analyze” function was used to report the tissue area positive for AT8 or Iba-1. The AT8 or Iba-1-positive area was expressed as a percentage of the brain tissue analyzed area. The stain positive area of each brain region (cortex, hippocampus, entorhinal cortex, and amygdala) was averaged for each mouse to give the ‘overall’ stain positive area. For microglia number quantification, each Iba-1-stained image was manually read to count the number of microglia per image using NIH ImageJ. For each brain region, the microglia counts were summed to give the total number of microglia per region per mouse. The overall number of microglia represents the sum of the microglia in each brain region per mouse. Additionally, microglial soma size in pixel units was determined in Iba-1-stained images of each region using NIH ImageJ by converting images to 8-bit, inverting to grayscale, and using the despeckle function to highlight the soma in the images. A threshold setting of > 10-pixel units was used to eliminate background signal detection. The sum of the microglia from all the brain regions was determined for each mouse to calculate the total number of microglial < 50- and > 50-pixel units per mouse. All the NIH ImageJ analysis was performed by two readers blinded to the experimental groups.

### Hippocampal neuronal loss quantification

Hippocampal neuronal loss was determined in six mice per treatment group (3 mice per sex). Three 20 µm sagittal mouse brain sections (600 µm apart and adjacent to the sections selected for AT8 immunostaining) per mouse were mounted on positive glass slides and allowed to dry overnight. Hematoxylin and Eosin (H&E) staining was performed by consecutive dips in acetal staining jars (Simport, Quebec, Canada) as follows: 30 s in distilled water, 10 min in filtered Mayer’s Hematoxylin (Fisher Scientific, MA, USA), 30 s in tap water, 30 s in Scott’s tap water/bluing reagent (10 g anhydrous MgSO_4_, 2 g NaHCO_3_, 1L tap water), 30 s in tap water, 4 min in Eosin Y (0.5% aqueous solution; Sigma Aldrich, MO, USA), 1 min in 95% ethanol, followed by 1 min in 100% ethanol. The slides received a final dip in xylene (Sigma Aldrich, MO, USA). Slides were cover-slipped with Permount mounting media (Fisher Scientific, MA, USA) and imaged using a light microscope (Motic, British Columbia, Canada). Different hippocampal regions (Dentate Gyrus (DG), CA1, CA2, and CA3) were imaged at a 10 × magnification, and the area occupied by the H&E-stained hippocampal neurons (µm^2^) of the granule cell layer in the DG and pyramidal layer in the CA1-3 regions was manually outlined and quantified using NIH ImageJ (version 1.53e, MD, USA) by a reader blinded to the experimental groups. The total hippocampal neuronal cell area per section was obtained by adding the regional cell areas.

### Western blot

Protein was extracted from the left frozen hemi-brains using the radio-immunoprecipitation assay (RIPA) buffer with Pierce Protease Inhibitor (Thermo Fisher Scientific, MA, USA) for Western blot as previously described [44–46]. Briefly, pulverized brains were homogenized in 2 volumes of RIPA (25 mM Tris–HCl, pH 7.6, 150 mM NaCl, 1% NP-40, 1% sodium deoxycholate, 0.1% sodium dodecyl sulfate (SDS) with 5 mM EDTA) and centrifuged at 20,800 × g for 20 min at 4 °C. The supernatants were collected and processed with 4 × Laemmli buffer (Bio-Rad, CA, USA) with 10% beta-mercaptoethanol followed by boiling for 10 min. Protein samples (30–50 μg) were separated on 4–20% SDS-ready precast gels (Bio-Rad, CA, USA) and transferred to polyvinylidene fluoride membranes (Bio-Rad, CA, USA). Membranes were sequentially subjected to blocking in Tris-buffered saline (TBS) containing 5% non-fat milk for 1 h at room temperature and probed with the mouse anti-PSD95 primary antibody (a marker of synaptic/neuronal health) (1:1000 in 3% non-fat milk, Santa Cruz Biotechnology, TX, USA) overnight at 4 °C followed by washing with TBS with 0.1% Tween-20 (TBST). Membranes were probed with an anti-mouse IgG kappa, HRP-linked secondary antibody (1:1000 in TBS containing 3% non-fat milk, Santa Cruz Biotechnology, TX, USA) for 1 h at room temperature followed by washing with TBST. Enhanced chemiluminescent (ECL) substrate reagent (Thermo Fisher Scientific, MA, USA) was used for secondary antibody detection and chemiluminescence was imaged using the Azure C500 gel imager (Azure Biosystems, CA, USA). Membranes were probed with an anti-β-actin antibody (1:1000 in TBS containing 3% non-fat milk, Santa Cruz Biotechnology, TX, USA) as a loading control, and membranes were incubated in the secondary antibody and ECL substrate as described above. NIH ImageJ (version 1.53e, MD, USA) was used to quantify the intensity of the Western blot bands and all the values were normalized to PS19-Saline mice.

### Pharmacokinetic (PK) study

All animal procedures were approved by the University of La Verne and Pomona College Institutional Animal Care and Use Committee. Eight-week-old male C57BL/6J mice (24 − 27 g) were obtained from the Jackson Laboratories (ME, USA). Mice had constant access to food and water and were maintained on a 12 h light/12 h dark cycle, and mice were randomly assigned to treatment groups. For the single-dose PK study, TfRMAb-TNFR fusion protein (1.75 mg/kg) or etanercept (0.875 mg/kg) were injected via the IP route in the volume of 130 − 140 μL per mouse. Each treatment group comprised 4 − 6 mice. Blood was collected in sodium citrate (9 parts blood and 1-part sodium citrate) at 3, 6, 20, and 24 h following IP administration, and plasma was collected.

For chronic dosing PK study, male heterozygous APP/PS1 mutant mice (strain B6C3-Tg APPswe, PSEN1dE9, 85Dbo/Mmjax, stock 004462, Jackson Laboratories, ME, USA) (~ 19 months of age) were injected IP three days per week for four weeks with either 1.75 mg/kg TfRMAb-TNFR (*n* = 4) or an equivalent volume of saline (*n* = 6). One week after 4-week dosing, all mice that were chronically treated with saline or TfRMAb-TNFR received a final single 1.75 mg/kg dose of the TfRMAb-TNFR fusion protein via the IP route to determine the impact of chronic TfRMAb-TNFR dosing on plasma PK. Blood was collected at 3, 6, and 24 h after the final injection for plasma collection. For the single and chronic PK studies, plasma TfRMAb-TNFR or etanercept concentrations were determined using a TNFR sandwich ELISA described below. Plasma concentration–time profiles were used to perform noncompartmental analysis to determine PK parameters (maximum plasma concentration (*C*_max_) and area under the plasma concentration − time curve (AUC)) using Thermo Scientific Kinetica 5.0 (Thermo Fisher Scientific, MA, USA), as described previously [47–49].

### TNFR ELISA

TfRMAb-TNFR fusion protein binding to TNF-α (Additional file 2: Fig. S1B), and TfRMAb-TNFR and etanercept plasma concentrations were quantified by a sandwich ELISA. The human TNF-α (hTNF-α) (PeproTech, Rocky Hill, NJ, USA) was the capture agent and 2 µg/mL of hTNF-α was plated in 96-well plates, and incubated overnight at 4 ºC. The wells were blocked with TBS containing 1% bovine serum albumin (TBSB) (0.01 M Tris/0.15 M NaCl/1% BSA/pH7.4) for 30 min at room temperature. Plasma samples (diluted 1:10 in TBSB) were added to the wells and incubated for 1 h at room temperature followed by washing with TBST. Alkaline phosphatase-conjugated detector agents, goat anti-human IgG-Fc fragment antibody (Bethyl, TX, USA) that binds to the human Fc domain of etanercept and goat anti-mouse light chain (kappa) antibody (Bethyl Laboratories, Inc., TX, USA) that binds to the TfRMAb domain of the TfRMAb-TNFR, were added to the wells for 1 h at room temperature followed by washing with TBST. Wells were then incubated with P-nitrophenyl phosphate solution (Sigma Aldrich, St. Louis, MO, USA) for 15 min in the dark at room temperature and the reaction was stopped by adding 1.2 M NaOH. Blank corrected absorbance measured at 405 nm was used to calculate plasma concentrations using a standard curve.

### Statistical analysis

All numerical variables are represented as mean ± SEM, and all statistical analysis was performed using GraphPad Prism 8 (GraphPad Software Inc., CA, USA) and as described by others [50]. Outliers were identified using the Grubb’s test. Independent two-sample *t*-test or one-way ANOVA with Holm–Sidak’s post hoc test was used to compare the means of two or more than two groups, respectively. To test the effects of two factors (e.g., treatment groups and time, or treatment groups and brain region), two-way repeated-measures ANOVA with Holm–Sidak’s multiple comparisons test was used. Open-field data were analyzed using two-way ANOVA with Holm–Sidak’s multiple comparisons test. Correlations between two numerical variables were analyzed by the Pearson correlation. A two-tailed *p* < 0.05 was considered statistically significant.

## Results

### Effect of biologic TNF-α inhibitors on pTau (Ser202, Thr205) in the PS19 mice

To determine the impact of TNF-α inhibitors on tau pathology, we quantified the pTau-positive area in the cortex, hippocampus, entorhinal cortex, and amygdala using the AT8 antibody. We then determined the overall AT8-positive area (average of the regional AT8-positive area). As expected, the overall AT8-positive area was significantly lower in the WT mice compared to the PS19-Saline controls, in both male (93% lower; *p* < 0.001) and female (78% lower; *p* < 0.01) mice (Fig. 1B–D; Additional file 3: Fig. S2A). Etanercept significantly reduced the overall AT8-positive area in both male and female PS19 mice by 73% (*p* < 0.01) and 58% (*p* < 0.05), respectively (Fig. 1B–D; Additional file 3: Fig. S2A). While TfRMAb-TNFR resulted in a 61% decrease (*p* < 0.05) in the overall AT8-positive area in the female PS19 mice (Fig. 1C–D; Additional file 3: Fig. S2A), surprisingly there was no change in the AT8-positive area in the TfRMAb-TNFR-treated male PS19 mice compared to the PS19-Saline mice (Fig. 1B, D; Additional file 3: Fig. S2A).

### Effect of the biologic TNF-α inhibitors on microgliosis in the PS19 mice

**Fig. 2:**
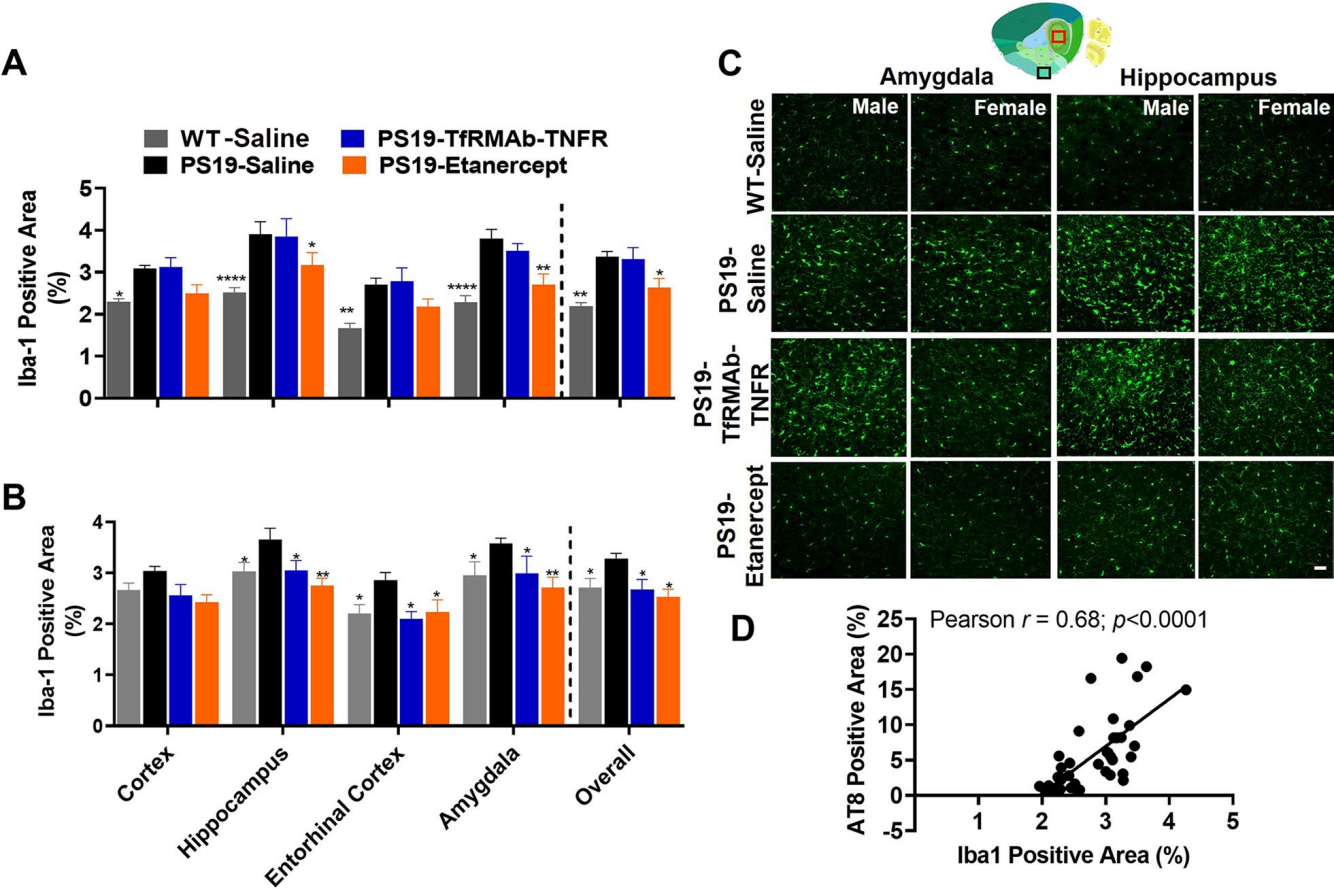
Effect of TNF-α inhibitors on microgliosis in the PS19 mice. Iba-1-positive area in different brain regions in male (**A**) and female (**B**) mice. The overall Iba-1-positive area, which is an average of the regional Iba-1-positive area was significantly reduced by etanercept in both sexes and by TfRMAb-TNFR in female mice (**A**,** B**). Representative images of Iba-1-positive microglia in the amygdala (black boxed region representing the piriform-amygdalar region in the thumbnail brain section image) and the hippocampus (red boxed region in the thumbnail brain section image) of the PS19 mice (**C**). Scale bar = 50 μm and thumbnail image in **C** was taken from the Allen Institute. Scatter plot showing a strong positive correlation between the overall AT8-positive area (%) and the overall Iba-1-positive area (%) in males and female mice combined (**D**; Pearson *r* = 0.68, *p* < 0.0001). Data are presented as mean ± SEM of 10–12 mice per treatment group (5–7 per sex per group). Two-way ANOVA with repeated measures with Holm–Sidak’s post hoc test was used to compare to PS19-Saline controls. *p < 0.05, **p < 0.01, ****p < 0.0001

Microgliosis was assessed in the cortex, hippocampus, entorhinal cortex, and amygdala by measuring the brain tissue area positive for the microglial marker, Iba-1. The overall Iba-1-positive area, which is the average of the regional Iba-1-positive areas, was significantly lower in the WT mice compared with the PS19-Saline controls in both male (35% lower; *p* < 0.01) and female (18% lower; *p* < 0.05) mice (Fig. 2A–C; Additional file 4: Fig. S3). TfRMAb-TNFR treatment significantly (*p* < 0.05) reduced the Iba-1-positive area by 18% in the female PS19 mice compared to PS19-Saline controls (Fig. 2B–C; Additional file 4: Fig. S3). No significant change in the Iba-1-positive area was observed in the TfRMAb-TNFR-treated male PS19 mice (Fig. 2A–C; Additional file 4: Fig. S3). Etanercept significantly (*p* < 0.05) decreased the Iba-1-positive area in both male (24% decrease) and female (23% decrease) mice compared to sex-matched PS19-Saline controls (Fig. 2A–C; Additional file 4: Fig. S3). A strong significant positive correlation was observed in the overall AT8-positive and overall Iba-1-positive area in the current study (Pearson *r* = 0.68, *p* < 0.0001; Fig. 2D). To determine if the reduction in the Iba-1-positive area was due to a reduction in microglia number or size, we quantified the number of microglia and estimated the microglial soma size. We found a significant increase (*p* < 0.01) in the total number of microglia in the PS19-TfRMAb-TNFR male mice compared to the PS19-Saline male mice (Additional file 5: Fig. S4A), but no difference in the total number of microglia in the female mice (Additional file 5: Fig. S4B). With respect to the soma size, the number of microglia with a smaller soma size (< 50-pixel units) was also significantly higher (*p* < 0.01) in the PS19-TfRMAb-TNFR male mice compared to the PS19-Saline male mice (Additional file 5: Fig. S4C). The number of microglia with a larger soma (> 50-pixel units) was significantly higher (*p* < 0.0001) in the PS19 male mice compared to the WT male mice (Additional file 5: Fig. S4E). Etanercept (*p* < 0.0001) and TfRMAb-TNFR (*p* < 0.01) treatment significantly reduced the number of microglia with a larger soma in the male PS19 mice (Additional file 5: Fig. S4E). On the other hand, there was a significant reduction in the number of microglia with smaller soma size in the WT-Saline (*p* < 0.001), PS19-TfRMAb-TNFR (*p* < 0.001) and PS19-Etanercept (*p* < 0.01) female mice compared with PS19-Saline female mice (Additional file 5: Fig. S4D). The number of microglia with a larger soma size was unchanged in the female mice (Additional file 5: Fig. S4F).

### Effect of the biologic TNF-α inhibitors on neuronal health in the PS19 mice

**Fig. 3:**
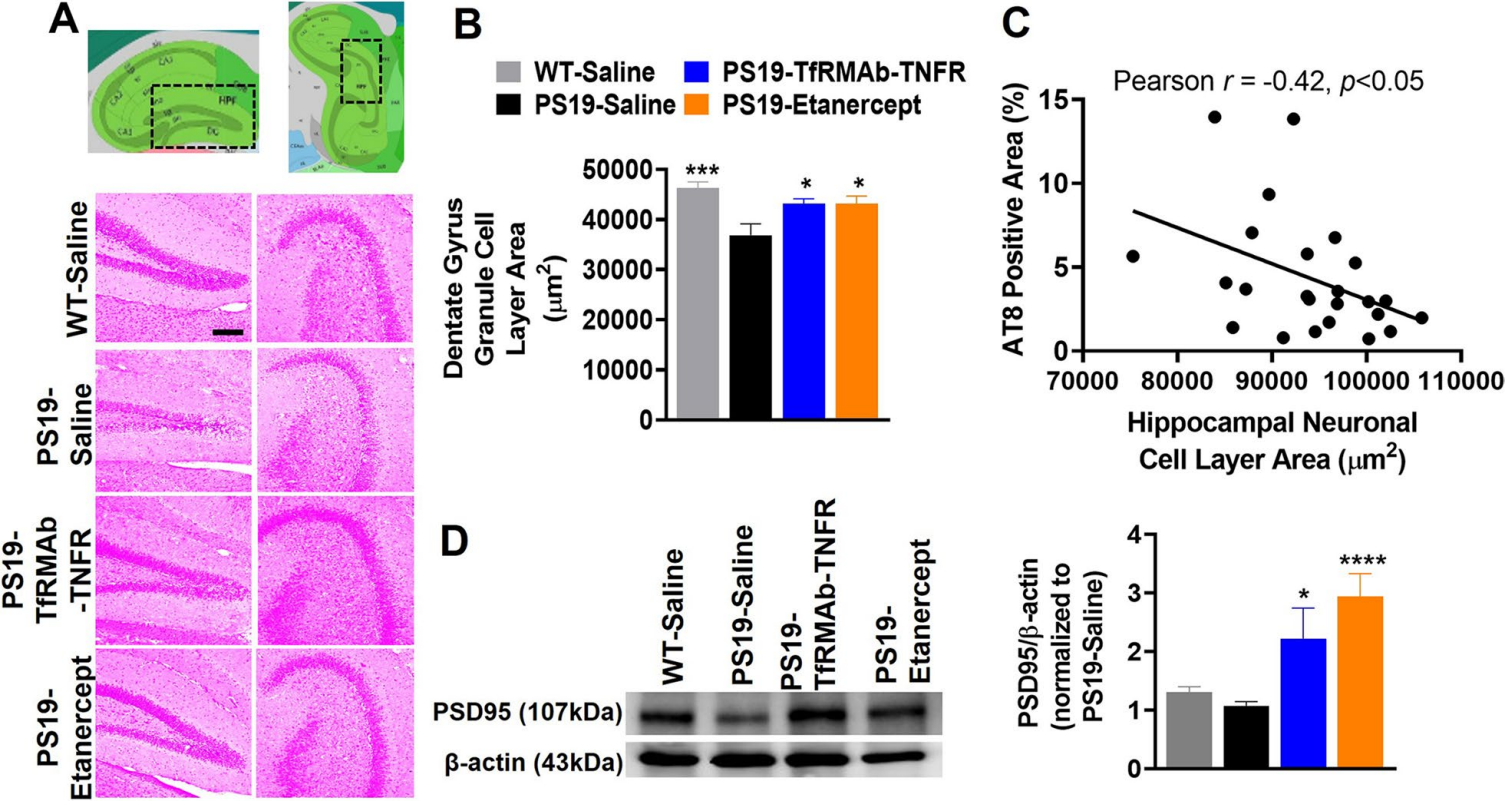
Effect of TNF-α inhibitors on neuronal health. Representative images of H&E-stained hippocampal granule cell layer of the dentate gyrus (DG) in the PS19 mice (**A**). Scale bar = 200 μm. The thumbnail images showing the DG regions that were imaged are taken and adapted from the Allen Institute. The DG granule cell layer area, as measured using ImageJ, was significantly greater in the WT-Saline, PS19-TfRMAb-TNFR, and PS19-Etanercept mice compared to PS19-Saline controls (**B**). Scatter plot showing that AT8-positive area (%) was negatively correlated with the total hippocampal neuronal cell area (**C**; Pearson *r* = -0.42, *p* < 0.05). PSD95 (synaptic/neuronal health marker) protein level in the whole-brain homogenates detected by Western blotting was significantly increased in the PS19-TfRMAb-TNFR and PS19-Etanercept mice compared to PS19-Saline controls (**D**). Data are presented as mean ± SEM of 6–11 mice per treatment group (3–7 per sex per group). For H&E staining, *n* = 6 mice per treatment group. For the PSD95 Western, *n* = 7–11 mice per treatment group. Male and female mice were combined due to a lack of sex-related effects. One-way ANOVA with Holm–Sidak’s post hoc test was used to compare to PS19-Saline controls. *p < 0.05, ***p < 0.001, and ****p < 0.0001

The area occupied by the hippocampal granule cell layer of the DG was significantly smaller (*p* < 0.001) in the PS19-saline mice compared with the WT mice (Fig. 3A, B). The loss of the DG granule cell layer in the PS19 mice was significantly attenuated (*p* < 0.05) by both etanercept and TfRMAb-TNFR treatment (Fig. 3A, B). No significant difference was observed in the hippocampal pyramidal cell layer area of the CA1-3 regions between the experimental groups (data not shown). A small but significant negative correlation was observed in the overall AT8-positive and the total hippocampal neuronal cell area (Pearson *r* = − 0.42, *p* < 0.05; Fig. 3C). The protein levels of the post-synaptic marker, PSD95 (a marker of synaptic health), in whole-brain homogenates, were not significantly reduced in the PS19-Saline controls (Fig. 3D). However, TfRMAb-TNFR and etanercept treatment led to a significant increase in the protein levels of PSD95 in the whole-brain homogenates of PS19 mice (Fig. 3D).

### Effect of TNF-α inhibitors on locomotion hyperactivity

**Fig. 4:**
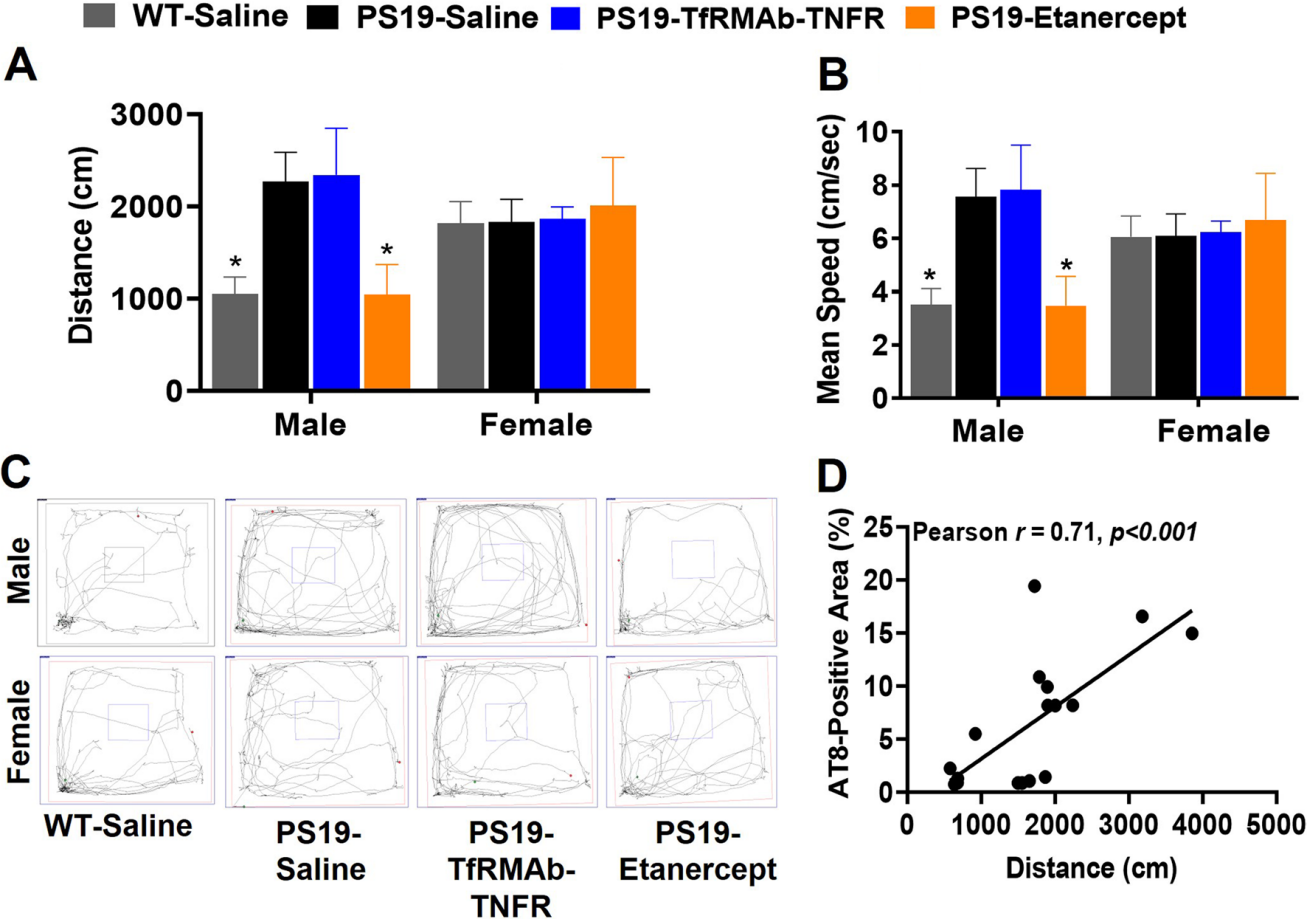
Effect of TNF-α inhibitors on locomotion. Significantly elevated locomotion indices, including distance travelled (**A**) and mean speed (**B)**, in the PS19 male mice compared to the WT male mice. Chronic etanercept treatment attenuated these changes in locomotion in the PS19 male mice. Chronic TfRMAb-TNFR treatment did not have any effect on locomotion in the male mice. No significant difference was observed in locomotion between any experimental groups in the female mice. Representative trajectory maps are shown in **C**. Distance travelled and overall brain AT8-positive area showed a strong significant positive correlation in the male mice (**D;** Pearson *r* = 0.71, *p* < 0.001). Two-way ANOVA with Holm Sidak’s post hoc test was used to compare with PS19-Saline (*n* = 5–7 mice per group per sex) in A and B. *p < 0.05 compared to PS19-Saline controls

The male PS19-Saline controls traveled a greater distance (*p* < 0.05) (Fig. 4A, C) and had a higher mean speed (*p* < 0.05) (Fig. 4B, C) compared to the WT male mice. Interestingly, this increase in distance traveled and mean speed was normalized by chronic etanercept treatment in the PS19 male mice, but not by chronic treatment with TfRMAb-TNFR (Fig. 4A–C). Neither the genotype nor the treatment affected locomotion hyperactivity in the female mice (Fig. 4A–C). A strong significant positive correlation was observed in the overall AT8-positive area in the brain and distance traveled in the male mice (Pearson *r* = 0.71, *p* < 0.001; Fig. 4D). No significant difference was observed in the time spent in the center, an indicator of anxiety-like behavior, between any experimental group in the current study (data not shown).

### Plasma PK after IP administration of biologic TNF-α inhibitors in mice

**Fig. 5:**
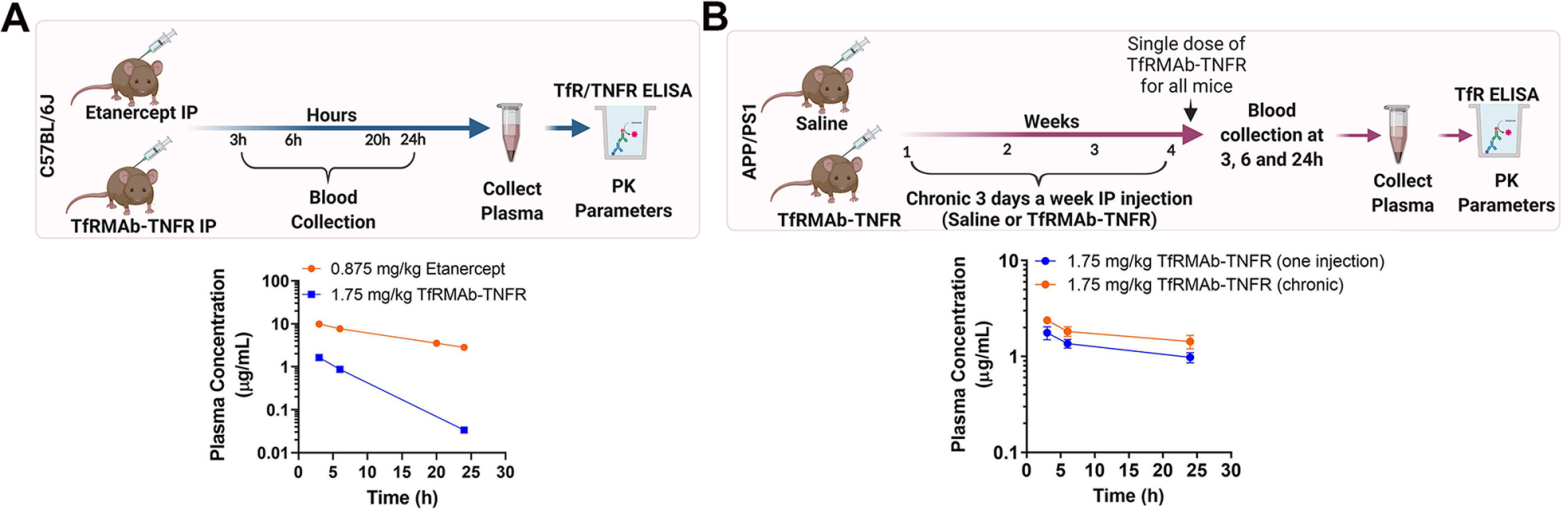
Plasma pharmacokinetic (PK) profile. Experimental design (top panel) and plasma concentration–time curves (bottom panel) following a single IP 1.75 mg/kg dose of TfRMAb-TNFR or IP 0.875 mg/kg dose of etanercept in C57BL/6 J mice (**A**). Experimental design (top panel) and plasma concentration–time curves (bottom panel) following chronic TfRMAb-TNFR dosing (**B**). Schematics in **A** and **B** were prepared using BioRender.com. For chronic TfRMAb-TNFR treatment, APP/PS1 mice were treated with a single 1.75 mg/kg IP injection after chronic 4-week treatment with saline (single injection group) or 1.75 mg/kg TfRMAb-TNFR (chronic injection group) and plasma samples were collected at 3 h, 6 h and 24 h following injection (**B**). Data are shown as the mean ± SEM of *n* = 4–6 mice per treatment group per time point

The plasma concentrations following a single IP administration of 1.75 mg/kg TfRMAb-TNFR or 0.875 mg/kg etanercept are shown in Fig. 5A, and plasma PK parameters are summarized in Table 1. The plasma C_max_ was 1.6 ± 0.08 µg/mL for the TfRMAb-TNFR fusion protein. The plasma C_max_ following an equimolar dose of etanercept was fivefold higher (*p* < 0.001) at 10.2 ± 1.3 µg/mL (Fig. 5A; Table 1). Accordingly, the plasma AUC over 24 h was eightfold higher (*p* < 0.001) for etanercept (7947 ± 664 µg•min/mL) compared to the TfRMAb-TNFR fusion protein (864 ± 46 µg min/mL) (Table 1).

**Table 1:** Plasma pharmacokinetic (PK) parameters following a single IP injection of 1.75 mg/kg TfRMAb-TNFR or 0.875 mg/kg etanercept, and following chronic IP injection of 1.75 mg/kg TfRMAb-TNFR (a single 1.75 mg/kg TfRMAb-TNFR injection after chronic 4-week treatment with saline or 1.75 mg/kg TfRMAb-TNFR)

Route	Group	Dose	Dosing frequency	AUC_(0–24 h)_	*C*_max_	AUC_(0–∞)_
		mg/kg		µg min/mL	µg/mL	µg min/mL
	Single injection PK					
IP	TfRMAb-TNFR	1.75	Once	864 ± 46	1.6 ± 0.08	875 ± 45
	Etanercept	0.875		7947 ± 664****	10.2 ± 1.3***	11,056 ± 843****
	Chronic dosing PK					
	TfRMAb-TNFR (single injection)	1.75	Once at the end of 4 weeks	1705 ± 157	1.8 ± 0.3	6114 ± 1268
	TfRMAb-TNFR (chronic injection)	1.75	3 days per week for 4 weeks	2352 ± 280	2.4 ± 0.2	6960 ± 2033

Data are shown as the mean ± SEM (*n* = 4–6 mice per treatment group per time point). Two-sample *t* test was used to compare PK parameters

***p < 0.001, ****p < 0.0001 compared to TfRMAb-TNFR for the single injection PK study.

To study the impact of chronic TfRMAb-TNFR dosing on plasma concentrations of the fusion protein, following 4-week chronic treatment with the TfRMAb-TNFR or saline, mice received a single 1.75 mg/kg IP injection of TfRMAb-TNFR and plasma samples were collected at 3 h, 6 h, and 24 h following injection. No significant differences were observed in the plasma PK parameters between the 4-week saline-treated mice receiving a single dose of 1.75 mg/kg TfRMAb-TNFR and 4-week TfRMAb-TNFR-treated mice receiving a single dose of the TfRMAb-TNFR (Fig. 5B; Table 1). The C_max_ values were comparable between the groups at 1.8 ± 0.3 µg/mL and 2.4 ± 0.2 µg/mL for the 4-week saline-treated mice receiving a single 1.75 mg/kg dose of TfRMAb-TNFR and 4-week TfRMAb-TNFR-treated mice receiving a single 1.75 mg/kg dose of TfRMAb-TNFR, respectively (Table 1). Similarly, the plasma AUC over 24 h was comparable between the two groups at 2352 ± 280 µg min/mL and 1705 ± 157 µg•min/mL for the 4-week saline-treated mice receiving a single 1.75 mg/kg dose of TfRMAb-TNFR and 4-week TfRMAb-TNFR-treated mice receiving a single 1.75 mg/kg dose of TfRMAb-TNFR, respectively (Table 1). It should be noted that the plasma concentrations of TfRMAb-TNFR following a single injection in Fig. 5A (blue curve) and Fig. 5B (blue curve) show differences that may be attributed to the different age and genotype of the mice used in Fig. 5A (8-week-old C57BL/6J) and Fig. 5B (19-month-old APP/PS1), but not to chronic saline dosing in Fig. 5B based on our previous work [44]. For this reason, the plasma concentrations following single and chronic TfRMAb-TNFR dosing were compared in two separate groups of 19-month-old APP/PS1 (in Fig. 5B; blue and orange curves). The single injection data presented in Fig. 5A (blue curve) was not compared with Fig. 5B (blue curve).

### Hematological profile

**Fig. 6:**
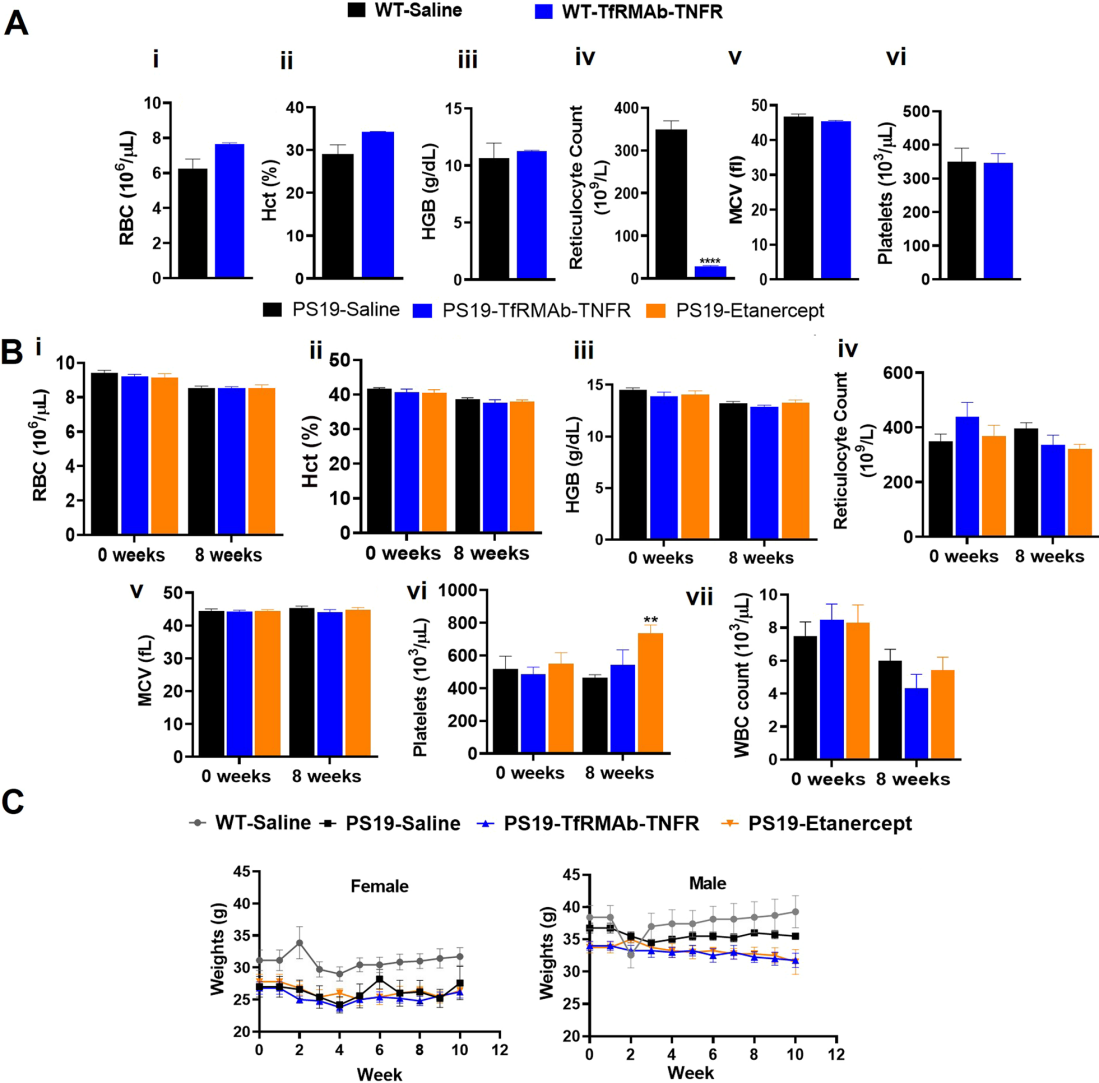
Complete blood count (CBC). A CBC was performed following a single 1.75 mg/kg TfRMAb-TNFR IP injection in male C57BL/6J mice (**A**) or chronic 8-week treatment with saline, 1.75 mg/kg TfRMAb-TNFR or 0.875 mg/kg etanercept in the male and female PS19 mice (**B**). For CBC analysis, red blood cell (RBC, **i**), hematocrit (Hct, **ii**), hemoglobin (HGB, **iii**), reticulocytes (**iv**), mean corpuscular volume (MCV, **v**), platelets (**vi**), and white blood cells (WBC) (**B, viii**) were measured. WBC counts were not measured following the single injection study. Sexes were combined for chronic CBC data due to no sex-related effects. The body weights of age-matched WT-Saline and PS19 mice were measured weekly. No significant difference was observed between the baseline and terminal body weights of the mice in any experimental group (**C**). For CBC data following a single injection (**A**), the two-sample *t* test was used to compare to the WT-Saline mice (*n* = 3 mice per group). Two-way repeated-measures ANOVA with Holm–Sidak’s post hoc test was used for CBC data after 8-week treatment to compare to PS19-Saline mice (*n* = 4–6 mice per group) (**B**) and body weight changes (*n* = 5–7 mice per group) (**C**). **p < 0.01, ****p < 0.0001

The hematologic profiles following a single 1.75 mg/kg TfRMAb-TNFR IP injection in C57BL/6 J mice and chronic 8-week treatment with 1.75 mg/kg TfRMAb-TNFR or 0.875 mg/kg etanercept in the PS19 mice are shown in Fig. 6A, B, respectively. A single injection of 1.75 mg/kg TfRMAb-TNFR induced a significant 92% reduction (*p* < 0.001) in reticulocyte counts compared to saline controls (Fig. 6A), while the other hematologic parameters remained unchanged (Fig. 6A). Interestingly, the suppression of reticulocyte count following a single injection normalized after chronic treatment with 1.75 mg/kg TfRMAb-TNFR for 8 weeks (Fig. 6B). Chronic treatment of TfRMAb-TNFR did not result in significant changes in other hematological parameters compared to PS19-Saline controls (Fig. 6B). Chronic 8-week treatment with 0.875 mg/kg of etanercept led to a significant 58% increase (*p* < 0.01) in the platelet counts compared to PS19-Saline controls, and no change in any other hematological parameter was observed (Fig. 6B). All the mice survived the duration of the study and compared to the baseline body weights, no significant change in the body weights of mice was observed throughout the study for any experimental group (Fig. 6C).

## Discussion

The results of the current study show that both the BBB-penetrating biologic TNF-α inhibitor (TfRMAb-TNFR) and the non-BBB-penetrating biologic TNF-α inhibitor (etanercept) exert protective effects in the female PS19 mice, with differential therapeutic effects in male PS19 mice. First, we show that etanercept significantly reduced pTau (ser202 and thr205) in both the male and female PS19 mice, and TfRMAb-TNFR reduced pTau in female PS19 mice. Second, pTau was significantly correlated with microgliosis, and etanercept significantly reduced microgliosis in both male and female PS19 mice. TfRMAb-TNFR reduced microgliosis in the female PS19 mice. Third, both TfRMAb-TNFR and etanercept improved neuronal health. Fourth, the locomotion hyperactivity of the PS19 male mice was attenuated with chronic etanercept treatment. Fifth, equimolar dosing of TfRMAb-TNFR and etanercept resulted in significantly different plasma concentrations, which may explain the differential therapeutic effects of these biologic TNF-α inhibitors in male mice. Finally, chronic 8-week treatment with these biologic TNF-α inhibitors had no major impact on the hematologic parameters of the PS19 mice.

The link between microglial activation-mediated neuroinflammation and tau pathology in AD and other tauopathies has been extensively studied [51, 52]. Accumulating research in this area postulates that microglial activation is an early event in tauopathies that precedes tau phosphorylation and tangle formation, eventually causing neuronal cell death and neurodegeneration [53, 54]. An important event in this cascade of microglia-mediated abnormal tau lesion accumulation is the release of cytokines that directly augment tau phosphorylation and the formation of tau tangles both in vivo and in vitro. For example, direct application of IL-1β or IL-6 to neuronal and microglial cultures increased tau phosphorylation and neuronal loss [55, 56]. Similarly, sustained brain levels of IL-1β and TNF-α increased tau phosphorylation and neuronal cell death in vivo [15, 57]. Accordingly, CNS TNF-α inhibition with the intracerebroventricular injection of a TNF-α antibody, and peripheral TNF-α modulation significantly reduced tau phosphorylation in a mouse models of amyloidosis [22, 58].

Consistent with these findings, we found a significant reduction in the AT8-positive immunoreactive area, which measures pTau at sites ser202 and thr205, with chronic treatment with both the biologic TNF-α inhibitors. We also observed sex differences in the efficacy of the two biologic TNF-α inhibitors in the current study. The BBB-penetrating TfRMAb-TNFR significantly reduced pTau in the female PS19 mice; interestingly, etanercept, which is shown not to cross the BBB [28], reduced pTau in both the male and female PS19 mice (Fig. 1; Additional file 3: Fig. S2). No change in total tau levels in the plasma and whole-brain homogenates was observed between the PS19-Saline and the biologic TNF-α inhibitor-treated PS19 mice (Additional file 3: Fig. S2). Further, the reduction in tau phosphorylation with the biologic TNF-α inhibitors paralleled the reduction in microgliosis in the current study, and the AT8-positive area showed a strong positive correlation with the Iba-1-positive area (Fig. 2). A recent report showed similar results wherein blockage of microglial proliferation with JNJ-40346527, a brain-permeant CSF1R inhibitor, reduced TNF-α and tau pathology in the spinal cords of the PS19 mice [59]. Together, these mouse studies reinforce the association between pTau lesions and microglial activation seen in human AD brains [51, 60] and in rodent models, including the PS19 mouse model used in the current study [31, 61].

One key characteristic of microglia is an activation-induced change in their morphology [62–64]. Under physiological conditions, microglia are said to be in a resting/quiescent/surveilling state that is typically characterized morphologically by the presence of highly ramified microglia with a small soma. However, once activated, the microglia transition into an amoeboid shape with an enlarged soma [62, 63], and soma size is a significant correlate of microgliosis [65]. Accordingly, the number of microglia with a larger soma size was significantly higher in the PS19 male mice compared to the WT mice suggesting the presence of more activated microglia in the PS19 male mice. The impact of the biologic TNF-α inhibitors on the microglial morphology varied by sex and the inhibitor. For example, the lack of change in microgliosis by TfRMAb-TNFR in the male mice (Fig. 2) appears to be driven by an increase in the number of microglia with a smaller soma size and an accompanying decrease in the number of microglia with a larger soma size (Additional file 5: Fig. S4). However, the reduction in microgliosis by etanercept appears to be driven by a reduction in the microglia with a larger soma size. In the female mice, the reduction in microgliosis appears to be driven by the reduction in the microglia with a smaller soma size rather than microglia with a larger soma size (Additional file 5: Fig. S4). Overall, our results suggest that the effect of the biologic TNF-α inhibitors on microgliosis is largely modulated by changes in microglia morphology rather than by changes in total microglia number. Future work using different markers for homeostatic (e.g., *P2ry12*) versus disease-associated (e.g., *Axl, Apoe4*) microglia will help elucidate the specific microglial phenotype modulated by the biologic TNF-α inhibitors [66].

Abnormal tau lesions correlate with neuronal loss and cognitive decline in AD [67], and a recent study showed that both tau and microglial activation can predict cognitive decline in AD [68]. In line with this, we wanted to determine if the attenuation in the pTau and microglial activation with the TNF-α inhibitors is associated with improvement or preservation of neuronal health. The PS19 mice exhibited a significant hippocampal neuronal loss in the DG, and we found a small but significant negative correlation between the AT8-positive area and hippocampal neuronal cell area in the PS19 mice (Fig. 3). These findings are consistent with the progressive hippocampal neuronal loss seen in the PS19 mouse model between 6- to 12-months of age [31]. We also measured the levels of PSD95, a post-synaptic protein that is widely used as a marker of synaptic health and is known to be modulated by TNF-α [69]. Notably, both the TfRMAb-TNFR and etanercept improved hippocampal and whole-brain tissue neuronal health in the current study (Fig. 3).

PS19 mice exhibit a hyperactive phenotype which is a characteristic of AD patients [39, 40]. Consistent with previous work with the PS19 mice [39], we found increased locomotion hyperactivity in the PS19 male mice (Fig. 4). Interestingly, the locomotion hyperactivity was associated with increased pTau lesions in the male mice in the current study, showing a direct relationship between these parameters in these mice. Locomotor hyperactivity was attenuated by chronic etanercept treatment but not TfRMAb-TNFR treatment, paralleling the reduction in AT8-positive tau lesions with these TNF-α inhibitors in the male mice. Though the PS19 mice display a hyperactive phenotype, these mice may also display deficits in hippocampal-dependent learning [39–41], and future work will investigate the effects of the biologic TNF-α inhibitors using different hippocampal-dependent memory testing paradigms.

Both TfRMAb-TNFR and etanercept can modulate peripheral TNF-α following systemic administration, while only the TfRMAb-TNFR can enter the brain to exert direct CNS anti-TNF-α effects [36]. Using radio-labeled TfRMAb-TNFR in mice, we have previously shown that a single IP injection of TfRMAb-TNFR for doses between 1–3 mg/kg results in a brain concentration of ~ 0.5% injected dose/gram brain at 24 h after injection [35]. Based on this, the predicted brain concentration of TfRMAb-TNFR following a single 1.75 mg/kg dose is 0.3 µg/g brain (2 nM) at 24 h following a single IP injection, assuming a 30 g mouse. The brain uptake of etanercept, on the other hand, is reported to be 20- to 13-fold lower than that of the brain-penetrating TNF-α inhibitor; furthermore, etanercept is found to distribute only to the brain blood volume, and not enter the brain parenchyma [28]. An alternate route for etanercept entry into the brain is via an impaired BBB in the PS19 mice. However, we did not observe changes in BBB tight junction proteins (ZO-1 and claudin-5) in the PS19 mice compared to the WT mice (Additional file 6: Fig. S5) consistent with previous work showing no change in BBB permeability in 8–10-month-old PS19 mice [70]. We measured TNF-α levels in the plasma and whole-brain homogenates using ELISA and Western blotting, respectively, but we did not see any significant change with the biologic TNF-α inhibitor treatment (Additional file 7: Fig. S6). However, we did see an increase in the whole-brain protein levels of IĸBα, which is degraded following TNF-α stimulation [71] and is therefore expected to increase after TNF-α inhibition, with TfRMAb-TNFR treatment (*p* < 0.01), and a similar trend was observed with etanercept treatment (*p* = 0.08; Additional file 7: Fig. S6). Taken together, the reduction in pTau, microgliosis, and improvement in neuronal health with both the TfRMAb-TNFR and etanercept in the current study indicates that these protective effects are modulated by peripheral, rather than direct CNS effects of these inhibitors in the PS19 mice. These findings are in agreement with recent reports showing that peripheral TNF-α alone can modulate microgliosis, neuronal health, and Aβ-dependent AD pathology in the 5xFAD mice [19, 20]. Accordingly, inhibition of peripheral TNF-α and modulation of the innate immune system were shown to reduce Aβ-associated tau-phosphorylation in the 3xTg mouse model [58].

Despite the ability of both TfRMAb-TNFR and etanercept to modulate peripheral TNF-α, we observed a significant difference in their therapeutic effects in the male PS19 mice. To elucidate the possible reason behind this difference in the male mice, we studied the plasma pharmacokinetics of these two inhibitors when given at equimolar doses to male mice. Furthermore, previous work with TfRMAb-based fusion proteins shows no difference in plasma pharmacokinetics between male and female mice [72]. Similarly, etanercept pharmacokinetics show only minor insignificant differences between males and females and were reported to remain largely consistent between sexes [73, 74]. Therefore, female mice were not used for plasma pharmacokinetic studies.

Etanercept is 50% TNFR and TfRMAb-TNFR is 25% TNFR based on amino acid sequence [36, 38]. As a result, the dose of etanercept was half that of TfRMAb-TNFR in the current study. However, despite equimolar dosing, the plasma *C*_max_ was > 500% higher and the plasma AUC was > 800% higher for etanercept compared to the TfRMAb-TNFR fusion protein after a single IP dose over 24 h (Fig. 5; Table 1). Plasma concentrations of TfRMAb-based therapeutics may be further altered following chronic dosing. For example, our recent work reported a significant increase in plasma clearance, and thereby a significant decrease in the plasma concentrations of TfRMAb (not fused to a therapeutic partner), following repeated administrations [47]. On the contrary, when the TfRMAb was fused to glial-derived neurotrophic factor (GDNF), no change in plasma clearance or concentrations of the TfRMAb-GDNF fusion protein was observed following chronic repeated dosing [72]. Based on this, we hypothesized that the plasma PK of TfRMAb-based fusion proteins is highly regulated by the fusion partner [47]. To determine the impact of chronic TfRMAb-TNFR dosing on plasma PK which may potentially impact the therapeutic efficacy of the fusion protein, we determined plasma concentrations of the fusion protein after repeated dosing. We found no change in the plasma concentrations of TfRMAb-TNFR following repeated dosing (Fig. 5; Table 1). Overall, the TfRMAb-TNFR fusion protein had a lower plasma concentration than etanercept, which is attributed to the TfR-mediated peripheral clearance of the TfRMAb-TNFR fusion protein from the circulation. The pTau load was 63% higher in the male mice compared to the female PS19 mice (Fig. 1), and it is conceivable that the higher pTau load seen in the male PS19 mice requires higher sustained plasma exposure of the TNF-α inhibitor, or a higher dose to produce a therapeutic effect. The low plasma exposure of TfRMAb-TNFR and the higher pTau load in the male PS19 mice may explain the lack of an effect of the TfRMAb-TNFR on pTau and microgliosis in the male PS19 mice in the current study. Notably, our previous work comparing the TfRMAb-TNFR and etanercept at a twofold higher dose (3 mg/kg) showed more robust effects of the TfRMAb-TNFR than etanercept in the male APP/PS1 mouse model of amyloidosis [42].

TfRMAb-based fusion proteins are associated with acute TfRMAb-mediated reticulocyte suppression, and TNF-α inhibitors including etanercept are potent immune modulators that can cause leukopenia and increase the risk of infections [47, 75, 76]. Accordingly, we found a significant reduction in reticulocytes with a single dose of the TfRMAb-TNFR, a TfRMAb-based therapy (Fig. 6). However, this suppression was acute and no reduction in reticulocytes or any other hematologic parameter was observed following 8-week chronic dosing of the TfRMAb-TNFR fusion protein. This is consistent with previous reports showing that reticulocyte suppression is short-lived and acute after administration of TfRMAb-based fusion proteins [44, 47, 75]. Mice treated with etanercept, on the other hand, had significantly elevated platelet counts following chronic dosing, which has been previously reported with etanercept use [77], with no other change in the hematologic parameters. Further, no change in weight along with a largely stable hematologic profile showed that chronic treatment with the biologic TNF-α inhibitors was safe in the PS19 mice [78].

The current study has some limitations. First, the absence of thioflavin-S-positive tau tangles (data not shown) in the brains of the 8-month-old PS19 mice in the current study precluded our ability to explore the effect of the biologic TNF-α inhibitors on tau aggregates. Second, we did not study the impact of these biologic TNF-α inhibitors on tau pathology and microgliosis in the locus coeruleus (LC), which is one of the first brain structures to develop tau pathology and is implicated in tau spreading [79]. A previous study showed accumulation of tau tangles in the LC of PS19 mice, a process that is highly variable and dependent on the motor deficits in these mice [79], and future work will be needed to elucidate the effects of these biologic TNF-α inhibitors on tau pathology and microgliosis in the LC. Third, we have only studied the effect of the TNF-α inhibitors on tau phosphorylated at ser202 and thr205; the effect on tau phosphorylation at other sites was not studied. Fourth, we used whole-brain homogenates to study the levels of PSD95, and we did not see a reduction in PSD95 levels in PS19 mice compared to WT mice. Since the hippocampus is the primary site of neuronal loss in the PS19 mice [31], the absence of a significant difference in the post-synaptic marker PSD95 between the PS19 and WT mice may be attributed to the use of whole-brain homogenate preparations in the current study rather than hippocampal brain homogenates to measure PSD95. Further, our data showing a reduction in microgliosis and pTau does not show causation. Whether the reduction in pTau reduces microgliosis or a reduction in microgliosis drives the reduction in pTau, is unclear. A recent study showed that amyloid potentiates microgliosis, which in turn drives tau pathology in the human AD brains [80]. Future work looking at the effects of these biologic TNF-α inhibitors in mouse models that combine both amyloid and tau pathology, along with the use of CSF1R inhibitors to deplete microglia, will allow us to elucidate the role of microglia-derived TNF-α in the protective effects of these biologic TNF-α inhibitors on both amyloid and tau pathology.

## Conclusion

**Fig. 7:**
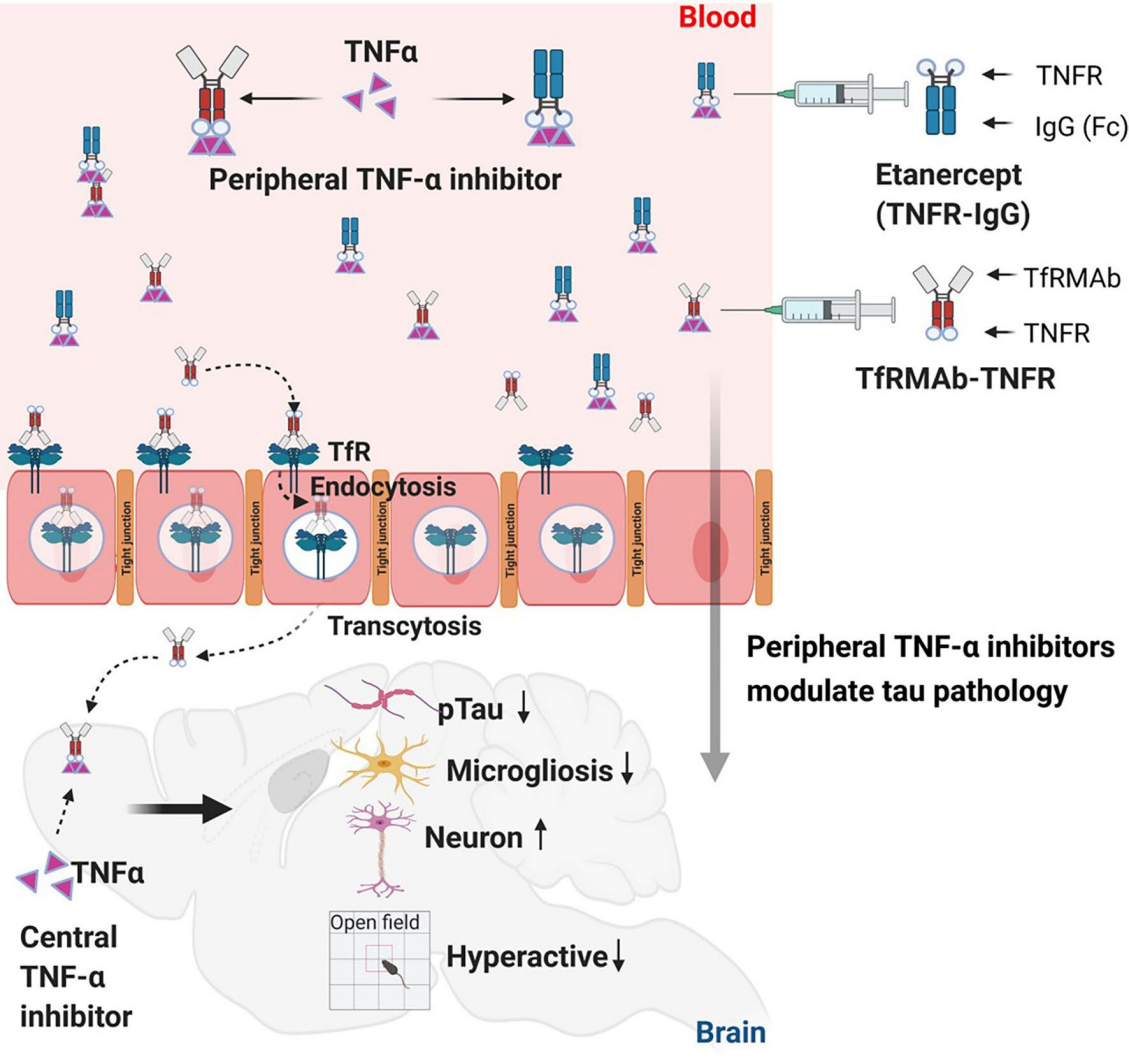
Systemic administration of etanercept (a non-BBB-penetrating TNF-α inhibitor) and TfRMAb-TNFR (a BBB-penetrating TNF-α inhibitor) results in a reduction in phosphorylated tau and microgliosis, and improvement in neuronal health in the PS19 mouse model of tauopathy. Etanercept treatment also reduces hyperactivity in the PS19 mice. These results support the development of biologic TNF-α inhibitors for tauopathies, including AD. Figure was prepared using BioRender.com

Overall, the data presented in this in vivo study in the PS19 mouse model of tauopathy are the first to report significant therapeutic effects of biologic TNF-α inhibitors on Aβ-independent tau pathology following systemic administration (Fig. 7). Our results show that both the BBB-penetrating TfRMAb-TNFR and non-BBB-penetrating TNF-α inhibitor modulate the microglia-tau-neurodegeneration axis, suggesting the involvement of peripheral TNF-α in these processes. Future work replicating these findings in other models of tauopathies and models combining Aβ and tau pathology may yield important preclinical data supporting the use of biologic TNF-α inhibitors for AD and other tauopathies.

## Supplementary Information

**Additional file 1: **Supplementary methods.

**Additional file 2**: **Fig. S1** High-affinity binding of the TfRMAb-TNFR to the TfR (A) and TNF-α (B). iPSC-derived brain endothelial cells (ihBMECs) cultured in Transwell inserts were treated with 1 µg/mL TNF-α and 7 µg/mL TfRMAb-TNFR for up to 3 days. TNF-α reduced TEER (C) and increased sodium fluorescein passage across the brain endothelial monolayer (D). These effects were normalized by TfRMAb-TNFR treatment. All the experiments were repeated three times independently and data were expressed as mean ± SEM. Two-way repeated-measures ANOVA or one-way ANOVA with Holm Sidak’s post hoc test was used in C and D, respectively. *p<0.05, ***p<0.001, ****p<0.0001. OD: optical density.

**Additional file 3:** **Fig. S2**. Representative AT8-stained images of the cortex and entorhinal cortex. Images were acquired at 40X. Scale bar = 30 µm (A). No significant change in the plasma (B) and brain total tau levels was detected using ELISA (C). A heat map showing the correlation between plasma total tau, brain total tau and overall AT8-positive area (%) in male and female mice combined (D). Total tau in plasma and brain shared a strong positive correlation (Pearson r = 0.76, p<0.0001). AT8-positive area showed a modest positive correlation with total tau in the plasma (Pearson r = 0.43, p<0.01) and brain (Pearson r = 0.46, p<0.05). Data are presented as mean ± SEM of n = 6-11 mice per treatment group in B and C. Male and female mice were combined due to a lack of sex-related effects. One-way ANOVA with Holm–Sidak’s post hoc test was used to compare to PS19-Saline controls in B and C. Pearson correlation was used for correlation analysis in D.

**Additional file 4: Fig. S3**. Representative Iba-1-stained images of the cortex and entorhinal cortex. Images were acquired at 10X with a digital 3X zoom. Scale bar = 25 µm.

**Additional file 5: Fig. S4.** Data from male mice are shown in A, C and E, and data from female mice are shown in B, D and F. A significantly higher number of total microglia were observed in PS19-TfRMAb-TNFR male mice compared to the PS19-Saline male mice (A). There was no significant difference in the total number of microglia in the female mice (B). The overall number of microglia is the sum of the microglia in the cortex, hippocampus, amygdala, and the entorhinal cortex. There was a significant increase in the overall number of microglia with a smaller soma size (< 50-pixel units) in the PS19-TfRMAb-TNFR male mice (C) and a significant decrease in the overall number of microglia with a larger soma size (soma size > 50-pixels) in the PS19-TfRMAb-TNFR, PS19-Etanercept, and WT-Saline male mice compared to PS19-Saline male mice (E). There was a significant decrease in the overall number of microglia with a smaller soma size (< 50-pixel units) in the PS19-TfRMAb-TNFR, PS19-Etanercept and WT-Saline female mice compared to the PS19-Saline female mice (D). There was no change in the number of microglia with a larger soma size (soma size > 50-pixels) in the female mice (F). Data are presented as mean ± SEM of n = 5-7 per treatment group. Two-way ANOVA with repeated measures with Holm–Sidak’s post hoc test was used to compare to PS19-Saline controls. *p<0.05, **p<0.01, ****p<0.0001.

**Additional file 6: Fig. S5**. The protein levels of the BBB tight junction proteins, ZO-1 (A) and claudin-5 (B), in whole-brain homogenates measured using Western blotting, were not significantly changed in the PS19-Saline controls compared to the WT-Saline group. Data are presented as mean ± SEM of n = 6-8 per treatment group. A Student’s t-test was used to compare the two groups.

**Additional file 7:** **Fig. S6.** Levels of TNF-α in the plasma (A) and whole-brain homogenates (B) were not significantly different between PS19-Saline and PS19-TfRMAb-TNFR and PS19-Etanercept mice, respectively. We attribute the lack of change in plasma and brain TNF-α levels with biologic TNF-α inhibitors to the time of plasma and brain sample collection. There was a 10-day lag between the last treatment dose (8 weeks after treatment initiation) and sample collection due to time for open-field testing (during week 9; Figure 1A). The plasma elimination half-lives of TfRMAb-TNFR and etanercept are ~ 4 h and ~13 h, respectively (Figure 5). Since it takes 6 elimination half-lives (24 h and 78 h for TfRMAb-TNFR and etanercept, respectively) for a drug to be completely eliminated from the blood circulation, we do not expect any circulating biologic TNF-α inhibitor at the time of sacrifice. Another potential reason for the lack of difference in plasma and brain TNF-α is the use of immunoassays. Since both the TfRMAb-TNFR and etanercept bind to TNF-α, detection of TNF-α using immunoassays may be complicated if the TNF-α is still bound to the biologic TNF-α inhibitor in the brain at the time of mouse sacrifice. Though this is unlikely considering the elimination half-lives of the drugs, to rule out any such interference, we measured the protein levels of IĸBα, which is degraded following TNF-α stimulation and is therefore expected to increase following TNF-α inhibition, in whole-brain homogenates using Western blotting (C). The TfRMAb-TNFR-treated PS19 mice had significantly higher (p<0.01) brain IĸBα (an indirect measure of the attenuation of TNF-α signaling). Similarly, PS19 mice treated with etanercept showed a trend (p=0.08) towards an increase in brain IĸBα compared to saline-treated PS19 mice, but this data did not reach statistical significance. Data are presented as mean ± SEM of n = 7-11 mice per treatment group. Male and female mice were combined due to a lack of sex-related effects. One-way ANOVA with Holm–Sidak’s post hoc test was used to compare to PS19-Saline controls. **p<0.01.

## Data Availability

The datasets used and/or analyzed during the current study are available from the corresponding author on reasonable request.

## Abbreviations

AD: Alzheimer’s disease
Aβ: Amyloid beta
BBB: Blood–brain barrier
BSA: Bovine serum albumin
CNS: Central nervous system
DG: Dentate Gyrus
GDNF: Glial-derived neurotrophic factor
Hct: Hematocrit
H&E: Hematoxylin and eosin
HGB: Hemoglobin
hTNF-α: Human TNF-α
MCV: Mean corpuscular volume
PFA: Paraformaldehyde
PK: Pharmacokinetic
PBS: Phosphate buffer saline
pTau: Phospho-tau
RIPA: Radio-immunoprecipitation assay
SDS: Sodium dodecyl sulfate
TNFIs: TNF-α inhibitors
TNFR: TNF-α receptor
TNFR-1: TNF-α receptor 1
TfRMAb: Transferrin receptor antibody
TX100: Triton X-100
TNF-α: Tumor necrosis factor-α
WBC: White blood cells
WT: Wild type
