# Supplementary material for: Biologic TNF-α inhibitors reduce microgliosis, neuronal loss, and tau phosphorylation in a transgenic mouse model of tauopathy
Source: J Neuroinflammation. 2021 Dec 31;18:312. doi: 10.1186/s12974-021-02332-7 (PMC8719395; doi:10.1186/s12974-021-02332-7)
Supplement: Supplementary file 1 — Additional file 1: Supplementary methods. [file 12974_2021_2332_MOESM1_ESM.docx]

**Supplementary Methods**

**Transferrin receptor (TfR) binding assay.** The binding affinity of the TfRMAb-TNFR fusion protein to TfR was conﬁrmed by ELISA. Nunc Maxisorp 96-well ELISA plates (Fisher Scientiﬁc, MA, USA) were coated with mouse TfR (200 ng/well, R&D system, MN, USA) overnight at 4 °C. After 1 h blocking with Tris-buﬀered saline (TBS) containing 1% bovine serum albumin (TBSB), TfRMAb-TNFR fusion protein at concentrations of 0.3−9000 ng/well was added for 1 h incubation, followed by three washes with TBS containing 0.05% Tween-20 (TBST). Wells were incubated with 400 ng/well of rabbit anti-human TNFRII antibody in TBSB (Sino Biologicals, Beijing, China) for 30 min at room temperature and then washed with TBST three times. Goat anti-rabbit IgG-H + L (GAR) secondary antibody−alkaline phosphatase (AP) conjugate (GAR-AP) (Vector Laboratories, CA, USA) was added into the wells for 45 min incubation. After washing with TBST, a signal was detected by adding P-nitrophenyl phosphate (Sigma Aldrich, MO, USA) for 30 min in the dark, and the reaction was stopped by adding 100 μL of 1.2 M NaOH per well. Absorbance (OD) was measured at 405 nm, and standard curves were used to determine maximal binding (Bmax) and dissociation constant (Kd) values (GraphPad Prism 8, CA, USA) as described previously (1).

**iPSC-derived human brain microvascular endothelial cell (ihBMEC) differentiation and treatment:** Human iPSC IMR90-4 line was differentiated into ihBMECs as described previously (2). Briefly, IMR90-4 cells (Wicell, WI, USA) were cultured on Matrigel (Corning, NY, USA)–coated surfaces in mTeSR-plus (STEMCELL Technologies, Vancouver, Canada). To initiate the differentiation, iPSCs were singularized at a density of 2.5 x 10^4^ cells/cm^2^ into 6-well plates. After a 3-day culture in mTeSR-plus, cells were cultured with 6 μM CHIR99021 (Selleckchem, TX, USA) in DMEM/Ham’s F12 (Thermo Fisher Scientific, MA, USA) with 1x MEM nonessential amino acids (Thermo Fisher Scientific, MA, USA), 0.5x GlutaMAX (Thermo Fisher Scientific, MA, USA), and 0.1 mM β-mercaptoethanol (Sigma Aldrich, MO, USA) for 1 day, followed by maintaining in DMEM/Ham’s F12 (Thermo Fisher Scientific, MA, USA) with 1x MEM nonessential amino acids (Thermo Fisher Scientific, MA, USA), 0.5x GlutaMAX (Thermo Fisher Scientific, MA, USA), and 0.1 mM β-mercaptoethanol (Sigma Aldrich, MO, USA) for 5 days. Cells were then cultured in human Endothelial Serum-Free Medium (hESFM, Invitrogen, CA, USA) supplemented with 20 ng/mL bFGF, 1x B27 (Thermo Fisher Scientific, MA, USA) and 10µM retinoic acid (Sigma Aldrich, MO, USA) for 3 days. The brain endothelial cells were re-plated at 1x10^6^ cells/cm^2^ in the same medium onto 24-well Transwell inserts (pore size 0.4 µm, Corning, NY, USA) coated with a mixture of collagen IV (400 μg/mL; Sigma Aldrich, MO, USA) and fibronectin (100 μg/mL; Sigma Aldrich, MO, USA). At 24 h after the re-plating, ihBMECs were cultured in hESFM with 1x B27 which was replaced daily for 3 days, followed by the treatment with 1 µg/mL human TNF-α (Biolegend, CA, USA) and 7 µg/mL TfRMAb-TNFR for 3 days.

**Transendothelial electrical resistance (TEER) measurement:** The permeability of the ihBMEC monolayer following the treatment with TNF-α and TfRMAb-TNFR was assessed by measuring the TEER using the EVOM2 Epithelial Volt/Ohm Meter and an STX-2 electrode system (World Precision Instruments LLC, FL, USA) daily. TEER of the ihBMEC monolayer was corrected for the TEER of empty transwells. TEER was expressed as % of baseline (i.e., before treatment TEER).

**Permeability of sodium fluorescein:** Sodium fluorescein (5 µg/mL) was added to the medium in the apical chamber. At 72 h following the treatment with TNF-α and TfRMAb-TNFR, medium in the basolateral chamber was collected, and mean fluorescent intensity (MFI) of sodium fluorescein was measured using a fluorescence plate reader (Molecular Devices, LLC, CA, USA) at excitation/emission wavelength of 460nm/515nm. Permeability coefficients were calculated as follows: Permeability coefficient (P, cm/s) = [volume of basolateral chamber / (surface area of transwell × the initial concentration of apical chamber)] × [the diffusion concentration of basolateral chamber / diffusion time] and was reported as % of control (untreated cells).

**Western Blot for TNF-α, IĸBα, ZO-1 and, claudin-5:** Protein was extracted from the left frozen hemi-brains using the RAB high salt buffer (G-Biosciences, MO, USA) with Pierce Protease Inhibitor (Thermo Fisher Scientific, MA, USA) for Western blot. Briefly, pulverized brains were homogenized in 2 volumes of RAB (0.1 M MES, 1 mM EGTA, 0.5 mM MgSO4, 0.75 M NaCl, 0.02 M NaF, 1 mM PMSF and 0.1% protease inhibitor mixture) and centrifuged at 20,800 x g for 40 min at 4 ˚C. The supernatants were collected and processed with 4× Laemmli buffer (Bio-Rad, CA, USA) with 10% beta-mercaptoethanol by boiling for 10 min. Protein samples (30-50 μg) were separated on 4-20% SDS-ready precast gels (Bio-Rad, CA, USA) and transferred to polyvinylidene fluoride membranes (Bio-Rad, CA, USA). Membranes were sequentially subjected to blocking in 5% non-fat milk in TBS for 1 h at room temperature and probed with the following primary antibodies (1:1000 in TBS containing 3% non-fat milk): rabbit anti-ZO-1 primary antibody (Thermo Fisher Scientific, MA, USA), the mouse anti-claudin-5 primary antibody (Santa Cruz Biotechnology, TX, USA), the mouse anti-IĸBα primary antibody (Cell Signaling Technology, MA, USA), and the rabbit anti-TNF-α primary antibody (Bio-Rad, CA, USA) respectively, overnight at 4 °C followed by washing with TBST. Membranes were probed with the following secondary antibodies (1:1000 in TBS containing 3% non-fat milk): HRP-linked anti-rabbit IgG (Cell Signaling Technology, MA, USA) or the HRP-linked anti-mouse IgG kappa (Santa Cruz Biotechnology, TX, USA) for 1 h at room temperature followed by washing with TBST. ECL substrate reagent (Thermo Fisher Scientific, MA, USA) was used for secondary antibody detection and chemiluminescence was imaged using the Azure C500 gel imager (Azure Biosystems, CA, USA). Equal protein loading amount was controlled by probing the membrane with an anti-β-actin antibody (1:1000 in 3% non-fat milk, Santa Cruz Biotechnology, TX, USA). NIH ImageJ (version 1.53e, MD, USA) was used to quantify the intensity of the Western blot bands and all the values were normalized to PS19-Saline mice.

**Total human tau ELISA:** Terminal plasma samples and RAB brain homogenates prepared above were diluted 300-fold and 100,000-fold, respectively, and used to detect human total tau using an ELISA as per the manufacturer’s instructions (KHB0041, Thermo Fisher Scientific, MA, USA).

**References**

1. Sun J, Boado RJ, Pardridge WM, Sumbria RK. Plasma Pharmacokinetics of High-Affinity Transferrin Receptor Antibody-Erythropoietin Fusion Protein is a Function of Effector Attenuation in Mice. Mol Pharm. 2019;16(8):3534-43.

2. Lippmann ES, Al-Ahmad A, Azarin SM, Palecek SP, Shusta EV. A retinoic acid-enhanced, multicellular human blood-brain barrier model derived from stem cell sources. Sci Rep. 2014;4:4160.
