# Supplementary figures and images for: Biologic TNF-α inhibitors reduce microgliosis, neuronal loss, and tau phosphorylation in a transgenic mouse model of tauopathy
Source: J Neuroinflammation. 2021 Dec 31;18:312. doi: 10.1186/s12974-021-02332-7 (PMC8719395; doi:10.1186/s12974-021-02332-7)

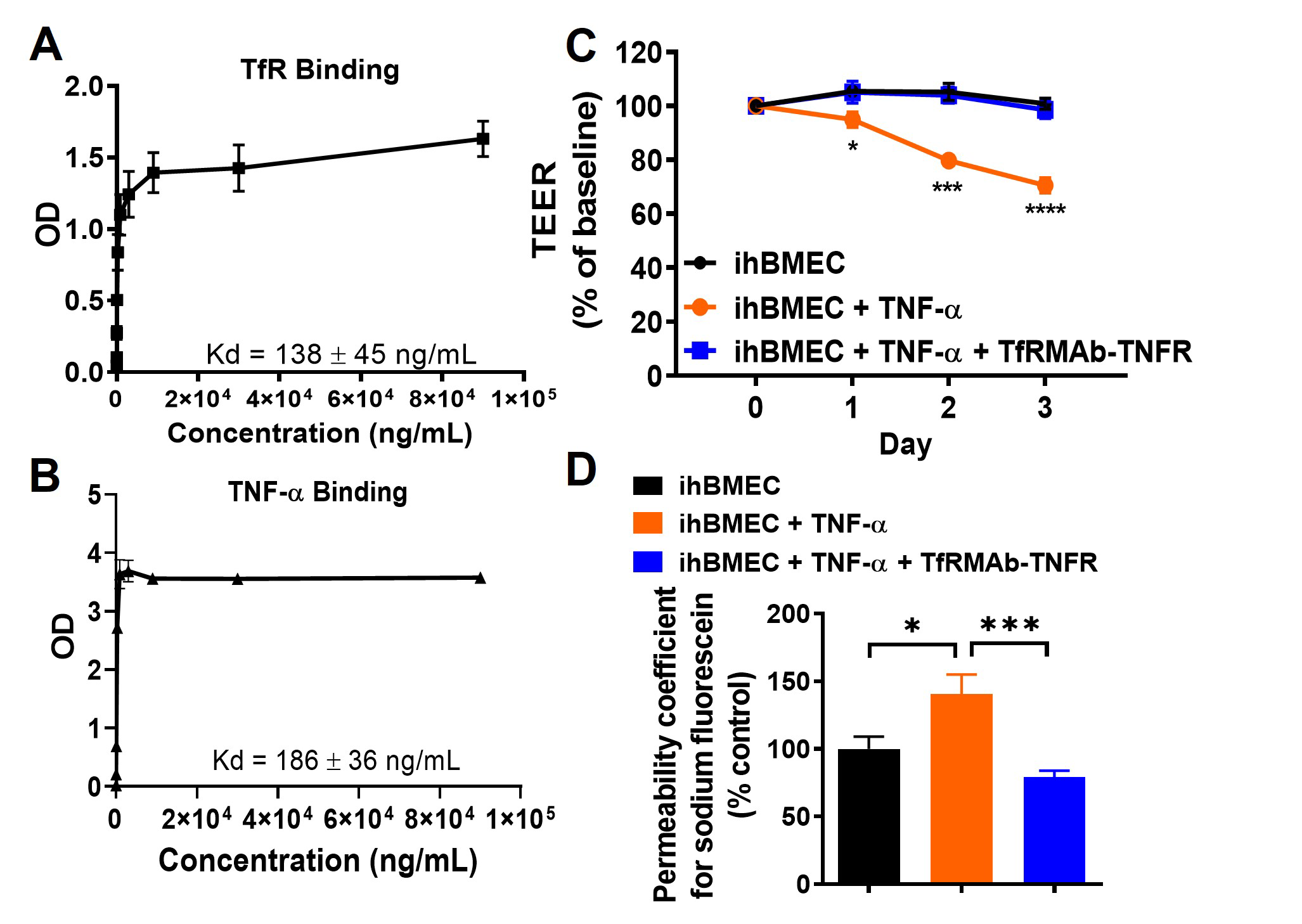

Supplement: Supplementary file 2 — Additional file 2: Fig. S1 High-affinity binding of the TfRMAb-TNFR to the TfR (A) and TNF-α (B). iPSC-derived brain endothelial cells (ihBMECs) cultured in Transwell inserts were treated with 1 µg/mL TNF-α and 7 µg/mL TfRMAb-TNFR for up to 3 days. TNF-α reduced TEER (C) and increased sodium fluorescein passage across the brain endothelial monolayer (D). These effects were normalized by TfRMAb-TNFR treatment. All the experiments were repeated three times independently and data were expressed as mean ± SEM. Two-way repeated-measures ANOVA or one-way ANOVA with Holm Sidak’s post hoc test was used in C and D, respectively. *p<0.05, ***p<0.001, ****p<0.0001. OD: optical density. [file 12974_2021_2332_MOESM2_ESM.tif]

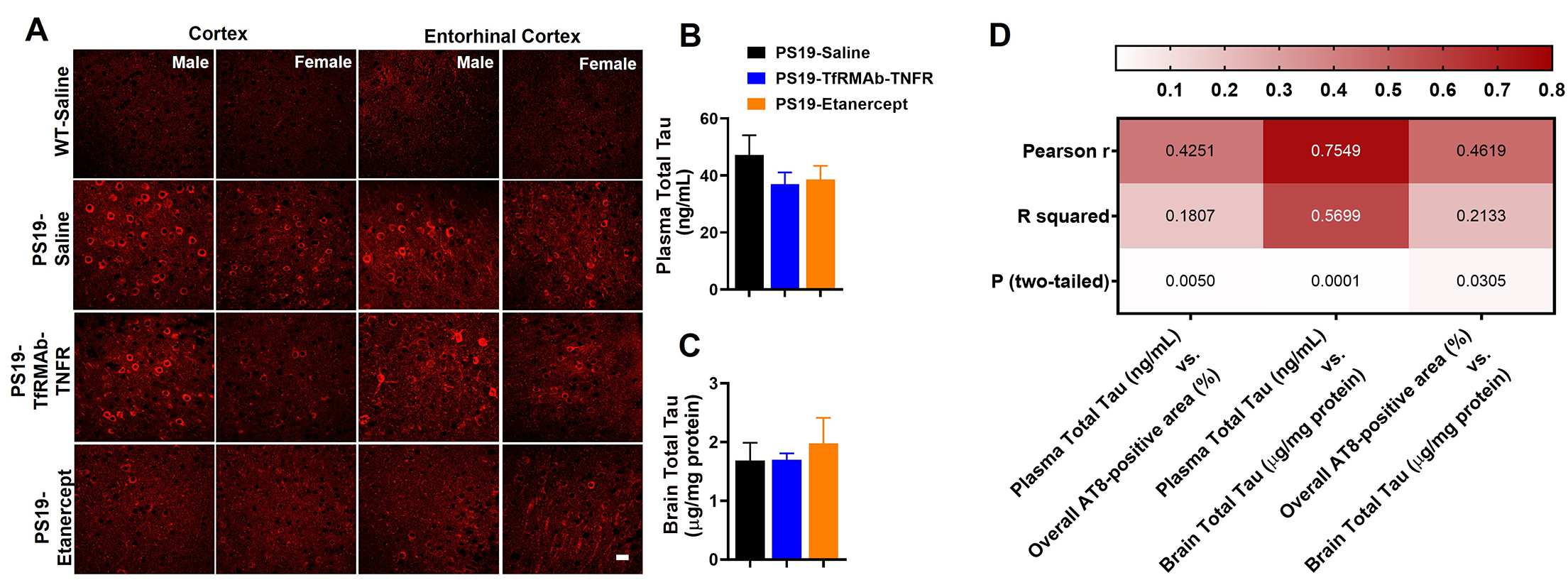

Supplement: Supplementary file 3 — Additional file 3: Fig. S2. Representative AT8-stained images of the cortex and entorhinal cortex. Images were acquired at 40X. Scale bar = 30 µm (A). No significant change in the plasma (B) and brain total tau levels was detected using ELISA (C). A heat map showing the correlation between plasma total tau, brain total tau and overall AT8-positive area (%) in male and female mice combined (D). Total tau in plasma and brain shared a strong positive correlation (Pearson r = 0.76, p<0.0001). AT8-positive area showed a modest positive correlation with total tau in the plasma (Pearson r = 0.43, p<0.01) and brain (Pearson r = 0.46, p<0.05). Data are presented as mean ± SEM of n = 6-11 mice per treatment group in B and C. Male and female mice were combined due to a lack of sex-related effects. One-way ANOVA with Holm–Sidak’s post hoc test was used to compare to PS19-Saline controls in B and C. Pearson correlation was used for correlation analysis in D. [file 12974_2021_2332_MOESM3_ESM.tif]

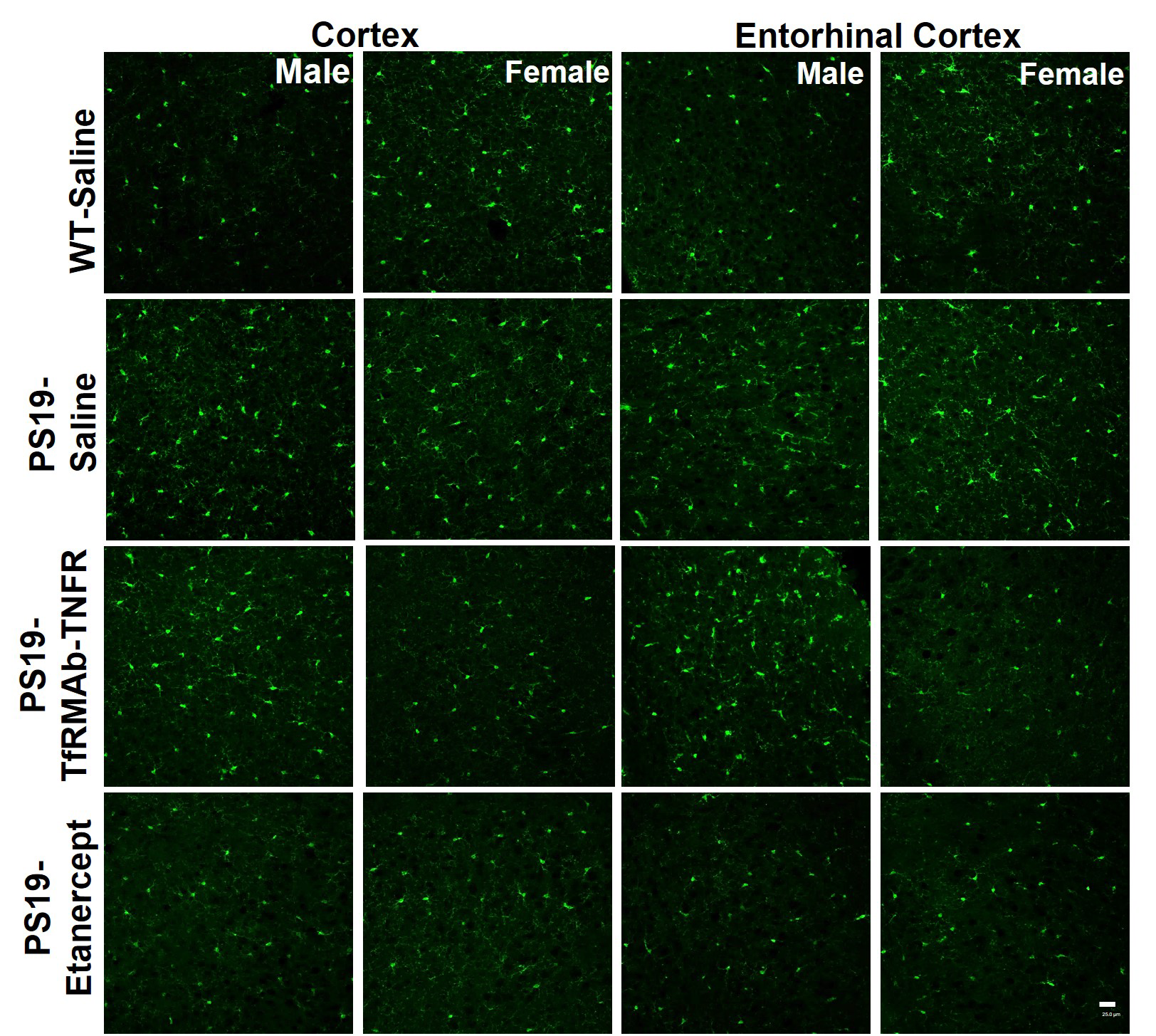

Supplement: Supplementary file 4 — Additional file 4: Fig. S3. Representative Iba-1-stained images of the cortex and entorhinal cortex. Images were acquired at 10X with a digital 3X zoom. Scale bar = 25 µm. [file 12974_2021_2332_MOESM4_ESM.tif]

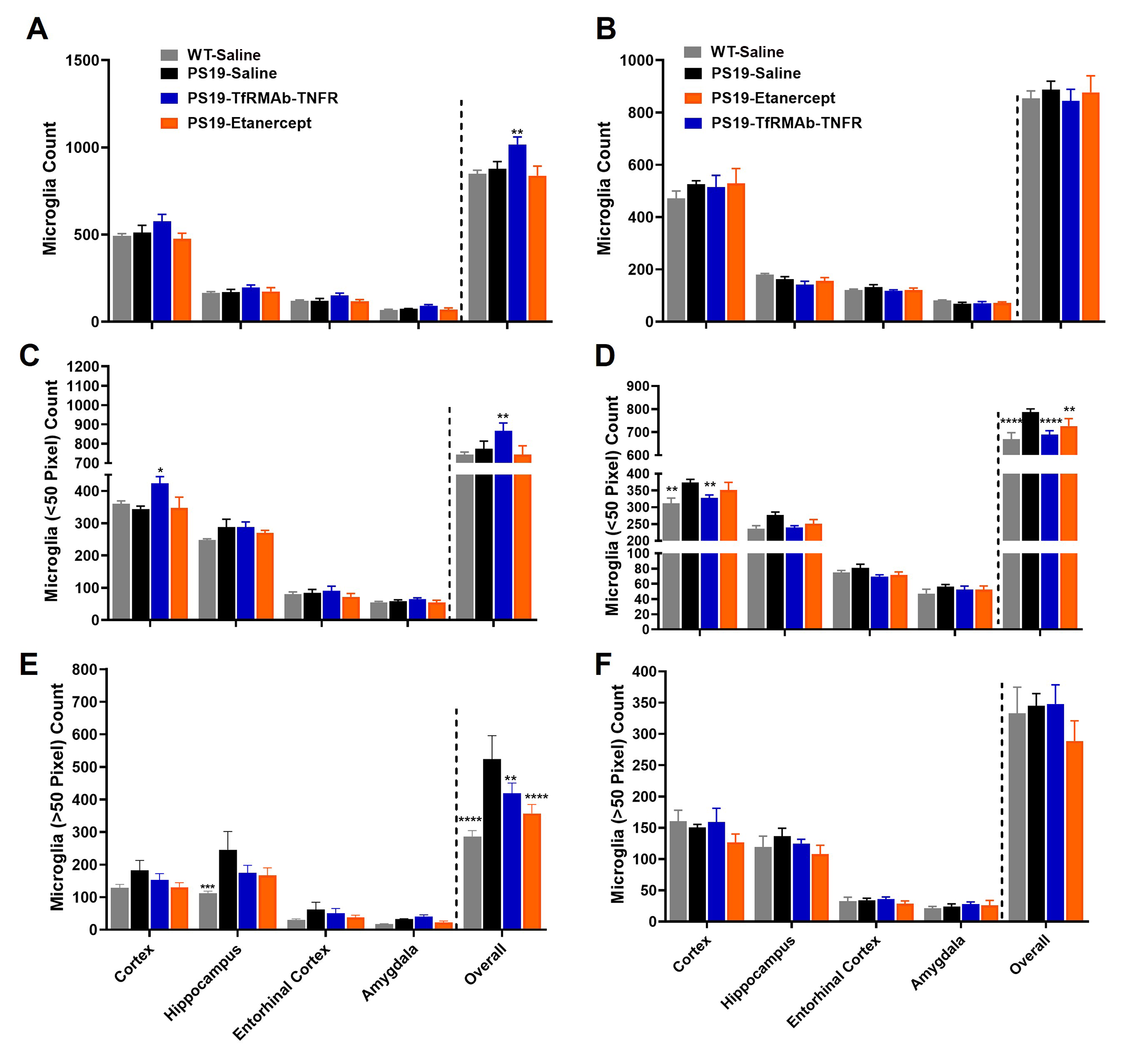

Supplement: Supplementary file 5 — Additional file 5: Fig. S4. Data from male mice are shown in A, C and E, and data from female mice are shown in B, D and F. A significantly higher number of total microglia were observed in PS19-TfRMAb-TNFR male mice compared to the PS19-Saline male mice (A). There was no significant difference in the total number of microglia in the female mice (B). The overall number of microglia is the sum of the microglia in the cortex, hippocampus, amygdala, and the entorhinal cortex. There was a significant increase in the overall number of microglia with a smaller soma size (< 50-pixel units) in the PS19-TfRMAb-TNFR male mice (C) and a significant decrease in the overall number of microglia with a larger soma size (soma size > 50-pixels) in the PS19-TfRMAb-TNFR, PS19-Etanercept, and WT-Saline male mice compared to PS19-Saline male mice (E). There was a significant decrease in the overall number of microglia with a smaller soma size (< 50-pixel units) in the PS19-TfRMAb-TNFR, PS19-Etanercept and WT-Saline female mice compared to the PS19-Saline female mice (D). There was no change in the number of microglia with a larger soma size (soma size > 50-pixels) in the female mice (F). Data are presented as mean ± SEM of n = 5-7 per treatment group. Two-way ANOVA with repeated measures with Holm–Sidak’s post hoc test was used to compare to PS19-Saline controls. *p<0.05, **p<0.01, ****p<0.0001. [file 12974_2021_2332_MOESM5_ESM.tif]

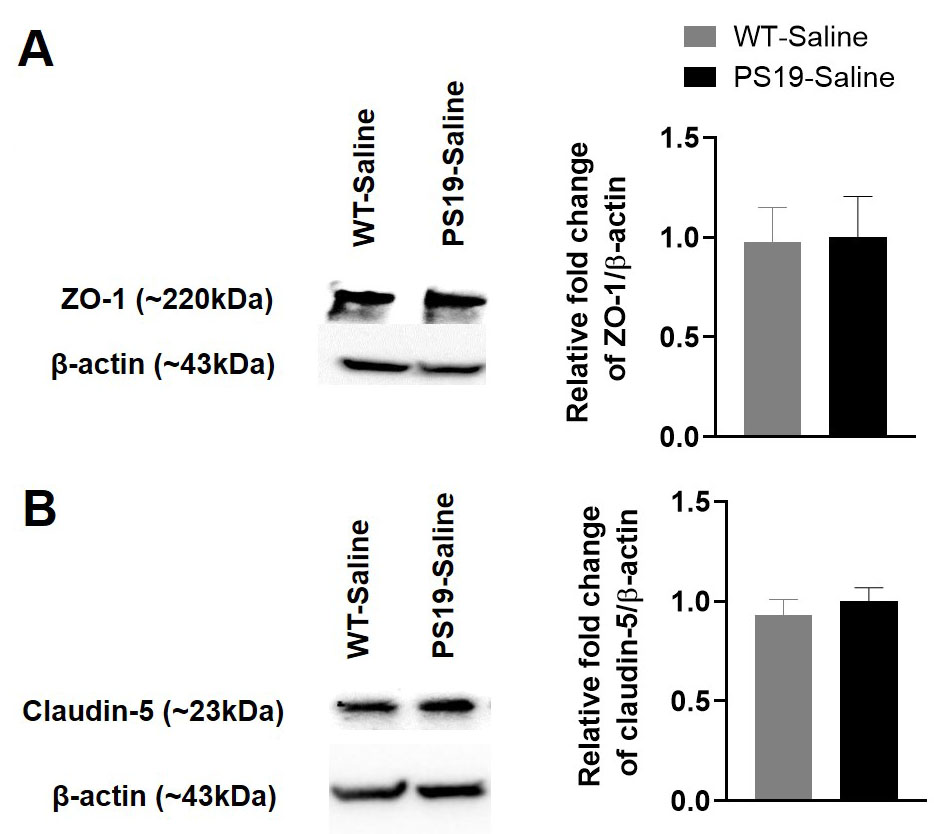

Supplement: Supplementary file 6 — Additional file 6: Fig. S5. The protein levels of the BBB tight junction proteins, ZO-1 (A) and claudin-5 (B), in whole-brain homogenates measured using Western blotting, were not significantly changed in the PS19-Saline controls compared to the WT-Saline group. Data are presented as mean ± SEM of n = 6-8 per treatment group. A Student’s t-test was used to compare the two groups. [file 12974_2021_2332_MOESM6_ESM.tif]

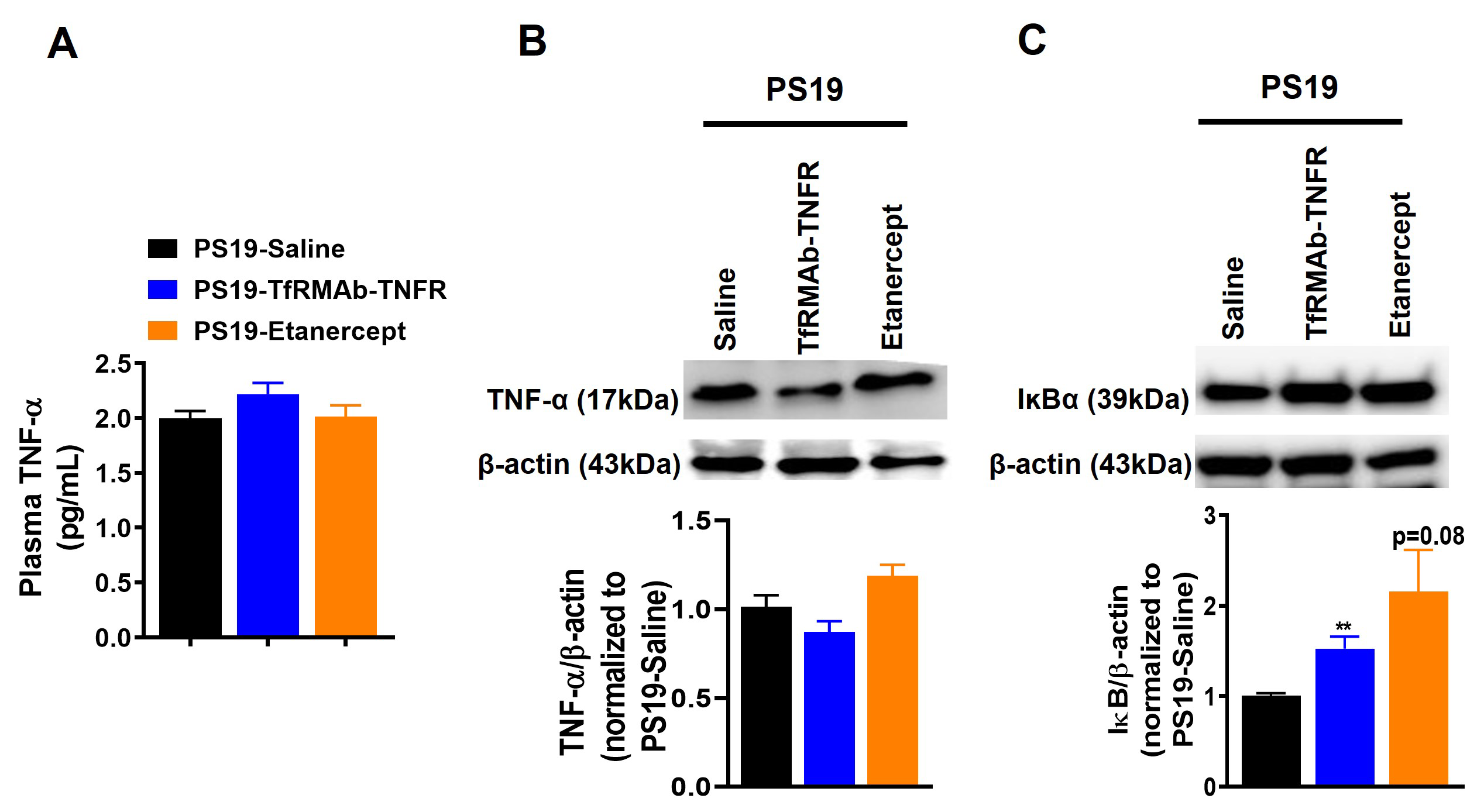

Supplement: Supplementary file 7 — Additional file 7: Fig. S6. Levels of TNF-α in the plasma (A) and whole-brain homogenates (B) were not significantly different between PS19-Saline and PS19-TfRMAb-TNFR and PS19-Etanercept mice, respectively. We attribute the lack of change in plasma and brain TNF-α levels with biologic TNF-α inhibitors to the time of plasma and brain sample collection. There was a 10-day lag between the last treatment dose (8 weeks after treatment initiation) and sample collection due to time for open-field testing (during week 9; Figure 1A). The plasma elimination half-lives of TfRMAb-TNFR and etanercept are ~ 4 h and ~13 h, respectively (Figure 5). Since it takes 6 elimination half-lives (24 h and 78 h for TfRMAb-TNFR and etanercept, respectively) for a drug to be completely eliminated from the blood circulation, we do not expect any circulating biologic TNF-α inhibitor at the time of sacrifice. Another potential reason for the lack of difference in plasma and brain TNF-α is the use of immunoassays. Since both the TfRMAb-TNFR and etanercept bind to TNF-α, detection of TNF-α using immunoassays may be complicated if the TNF-α is still bound to the biologic TNF-α inhibitor in the brain at the time of mouse sacrifice. Though this is unlikely considering the elimination half-lives of the drugs, to rule out any such interference, we measured the protein levels of IĸBα, which is degraded following TNF-α stimulation and is therefore expected to increase following TNF-α inhibition, in whole-brain homogenates using Western blotting (C). The TfRMAb-TNFR-treated PS19 mice had significantly higher (p<0.01) brain IĸBα (an indirect measure of the attenuation of TNF-α signaling). Similarly, PS19 mice treated with etanercept showed a trend (p=0.08) towards an increase in brain IĸBα compared to saline-treated PS19 mice, but this data did not reach statistical significance. Data are presented as mean ± SEM of n = 7-11 mice per treatment group. Male and female mice were combined due to a lack of sex- [file 12974_2021_2332_MOESM7_ESM.tif]
